# Lineage tracing of soma-to-primordial germ cell-like conversion in human tumor cell line

**DOI:** 10.1016/j.isci.2026.116214

**Published:** 2026-06-04

**Authors:** Jing Li, Fengyu Zhang, Ji Xiong, Aiping Liu, Peipei Liu, Zhan Ma, Hui-Kuan Lin, Chunfang Liu

**Affiliations:** 1 Department of Laboratory Medicine, Huashan Hospital, Shanghai Medical College, Fudan University, Shanghai 200040, China; 2 Department of Pathology, Huashan Hospital, Shanghai Medical College, Fudan University, Shanghai 200040, China; 3 Department of Laboratory Medicine, Shanghai Children’s Hospital, Shanghai Jiao Tong University, Shanghai 200040, China; 4 Department of Pathology, Duke University School of Medicine, Durham, NC 27710, USA

**Keywords:** Biological sciences

## Abstract

Identifying the origin of tumor-initiating ability is critical for targeted therapies. While early primordial germ cells (PGCs) inherently possess this ability, our previous study indicated that soma-to-PGC-like conversion (SPLC) facilitates its acquisition in mouse breast tumor cells. This study reports evidence of SPLC activation in the human hepatic tumor cell line HL7721, where tumor cells spontaneously exhibit sequential developmental stages, progressing from somatic tumor cells through intermediate stages to PGC-like and late germ cell-like stages. Knocking out PGC specification inducers (*ACVR1*, *SMAD5*, *PRDM1*, or *SOX17*) or fate-maintaining genes (*NANOG* or *DDX4*) suppressed both SPLC and tumor initiation. Collectively, our findings suggest that SPLC might be activated in human tumor cells, serving as a potential pathway for gaining tumor-initiating ability that involves human PGC-related pathways. These results demonstrate that the acquisition of PGC-like fate in somatic cells drives tumor malignant progression, providing a potential paradigm for targeted cancer therapy.

## Highlights

• Soma-to-primordial germ cell-like conversion is activated in human hepatic tumor cells
• The primordial germ cell-like subset acts as the core population for tumor initiation
• Primordial germ cell-related genes regulate soma-to-primordial germ cell-like conversion

## Graphical abstract

**Figure:**
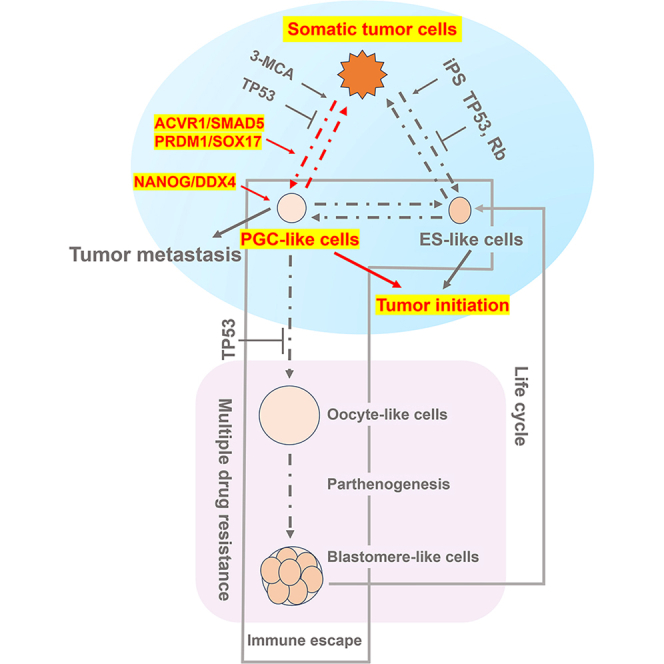

## Introduction

Tumor-initiating ability is essential for tumor relapse and metastasis.^1^ Identifying the origin of tumor-initiating ability is of great interest because it provides potential targets for therapy.^1^ Of note, while embryonic stem (ES) cells possess inherent tumor-initiating ability,^2^^,^^3^ early primordial germ cells (PGCs), as precursors of sperm and eggs, possess inherent abilities for both tumor initiation^3^^,^^4^ and migration^5^ at the early stage of development. Accumulating evidence uncovers that the embryonic/germ cell traits of tumors play crucial roles in malignant tumor behaviors.^6^^,^^7^^,^^8^^,^^9^^,^^10^^,^^11^^,^^12^^,^^13^ Interestingly, early reports and our previous studies have shown that the ES/PGC-like state exists in many types of tumor cell lines and might be reactivated in mammalian somatic cells by carcinogenic factors,^14^^,^^15^^,^^16^^,^^17^^,^^18^^,^^19^ such as chemical carcinogens and *TP53* deletion.^20^^,^^21^^,^^22^ Moreover, we previously reported that the PGC-like tumor cells play important roles in liver metastasis of mouse tumors and can establish a life cycle: they develop into oocyte-like tumor cells, subsequently generate blastomere-like tumor cells, and then return to somatic tumor cells, which contributes to multidrug resistance and tumor recurrence.^22^ Therefore, the PGC-like state in tumors could link various malignant traits of tumors together, and clarifying how tumors acquire the PGC-like state and tumor-initiating ability is essential to understanding tumor biology.

Based on the high similarities between germ cell development and tumor formation, the century-old embryonal/gametogenesis hypothesis of tumors was proposed and extended by Old et al., who suggested that reacquiring the PGC-like fate in somatic cells is a driving force for tumor malignant progression.^23^^,^^24^ Our previous study revealed that soma-to-PGC-like conversion (SPLC) can be activated and plays an essential role in acquiring tumor-initiating ability in the mouse breast tumor cell line 4T1. However, it remains unknown whether SPLC actually occurs and serves as one of the key mechanisms underlying tumor initiation in human tumors.

In the current study, HL7721 cells, derived from a single clone of the human hepatic cell line SMMC7721, were used as a model. Our findings suggest that SPLC may occur in human tumors, potentially serving as a mechanism for acquiring tumor-initiating ability that involves the human PGC (hPGC) specification pathway. Through lineage tracing and genetic knockout experiments, our results provide support for Old’s concept and offer insights into a sequence of molecular events underlying tumor-initiating ability acquisition, as well as potential therapeutic targets for human tumors.^23^^,^^24^

## Results

### Generation of germ cell-like cells from hepatic tumor cells

**Figure 1:**
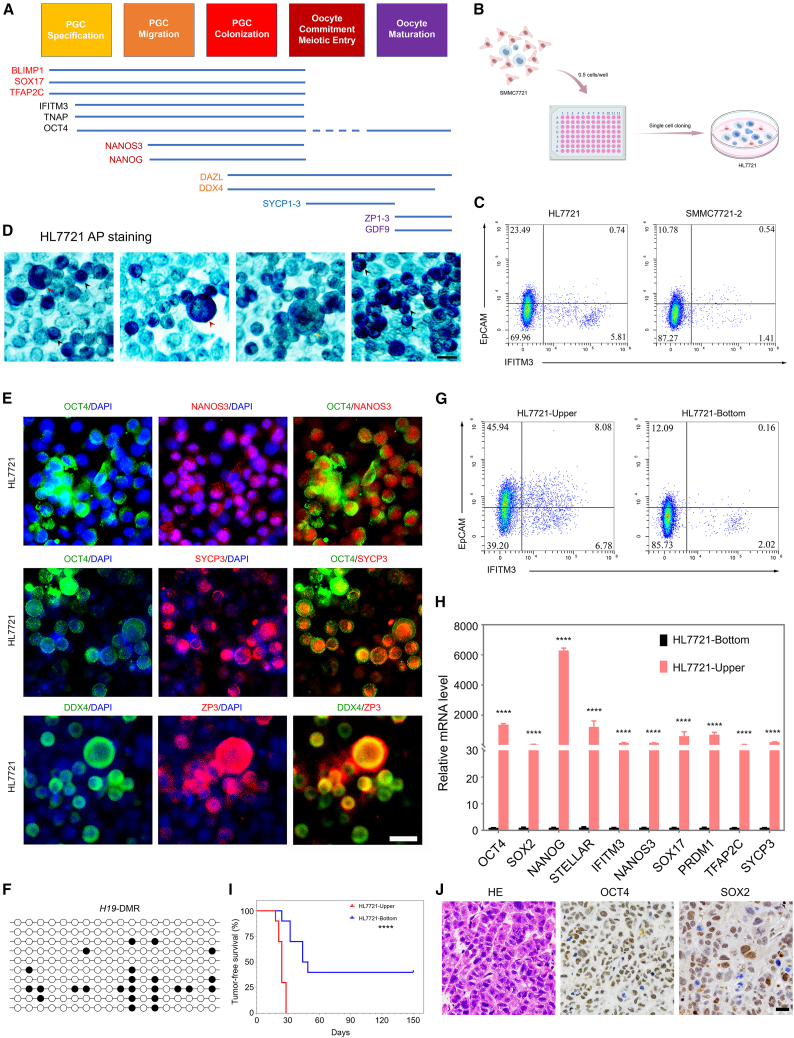
PGC-like cell formation in HL7721 cells (A) The sequential expression of specific genes in human PGC specification and development. (B) Derivation of the HL7721 from human hepatic SMMC7721 tumor cell line by the single-cell clone. (C) FCM showed the cells positive for IFITM3 and/or EPCAM in HL7721 and SMMC7721-2. (D) Bright-field images and AP staining showed the germ cell-like cells (PGC-like cells, black arrow; oocyte-like cells, red arrow) and blastomere-like cells (yellow arrow) at different developmental stages in HL7721 cultures. Scale bars, 25 μm. (E) Immunofluorescence analysis showing the expression of OCT4, NANOS3, DDX4, SYCP3, and ZP3 in the HL7721 cultures. Scale bars, 25 μm. (F) Sodium bisulfite sequencing revealed a hypomethylated state of the *H19* DMR in HL7721 cells (*n* = 10). Black circles represent methylated CpG sites, and white circles represent unmethylated CpG sites. (G) FCM analysis of cells positive for IFITM3 and/or EPCAM in the upper and bottom layers of the HL7721 cultures. (H) RT-qPCR analysis of the relative expression of germ cell development-related genes in the upper and bottom layers of HL7721 cultures (*n* = 3). Data are presented as mean ± SD and analyzed by unpaired *t* test. (I) The tumor-free survival curve evaluating the tumor initiation potential in the upper and bottom layers of the HL7721 cultures (*n* = 10). Data are analyzed by log rank test. (J) Representative images of xenograft tumor tissue sections with H&E staining and immunohistochemistry staining for OCT4 and SOX2 from HL7721 in mice. Scale bars, 25 μm. ∗*p* < 0.05, ∗∗*p* < 0.01, ∗∗∗*p* < 0.001, ∗∗∗∗*p* < 0.0001; n. s., no significant statistical difference.

The precise sequential expression of specific genes in hPGC specification and development is less clear than that in mouse PGCs.^25^ We summarized the likely sequential expression of specific genes in hPGC specification and development based on existing literature^25^^,^^26^^,^^27^^,^^28^^,^^29^ (Figure 1A). We first investigated the origin and tumorigenicity of the PGC-like cells. We selected a single-cell clone with a strong ability to generate PGC-like cells from the human hepatic SMMC7721 tumor cell line (named HL7721; Figure 1B), which was used as a model in the current study. The robust PGC-like cell generation capacity of HL7721 was validated by flow cytometry (FCM) through comparison with another single-cell clone, SMMC7721-2 (Figure 1C). We addressed whether germ cell-like cells could indeed be generated in HL7721 cultures, based on the documented traits of PGCs.^5^^,^^25^^,^^27^^,^^29^ Tissue-nonspecific alkaline phosphatase (TNAP), one of the earliest PGC and pluripotency markers in humans and mice,^5^^,^^30^^,^^31^ was used to identify the generation of PGC-like cells in HL7721 cultures. TNAP staining showed that PGC-like cells were round in morphology (∼10–15 μm in diameter), had a high nuclear-to-cytoplasmic (N/C) ratio, were strongly positive for TNAP, were generated spontaneously, and were often enriched in the upper layer and formed clusters in HL7721 cultures (Figures 1D and S1A), resembling cultured PGCs. Moreover, germ cell-like cells with distinct developmental stages were observed in HL7721 cultures (Figures 1D and S1A–S1C). Morphologically, late-stage germ cell-like cells were larger round cells (∼20–50 μm in diameter), resembled oocytes, and showed gradually decreased TNAP-positive staining (Figures 1D and S1A). In line with this, a subset of oocyte-like cells displayed a germinal-vesicle (GV)-like structure, a hallmark of oocyte development, and expressed OCT4, DDX4, and ZP3 (Figures 1E, S1A, and S1C). Occasional blastomere-like cells, also OCT4 and ZP3 positive, were observed within HL7721 cultures (Figures S1A and S1C). Together, these findings suggest the potential for PGC-like cells to develop into oocyte-like cells, further supporting their resemblance to endogenous PGCs. The emergence of oocyte-like cells from male cancer cells is, in fact, consistent with established tenets of developmental biology.^32^

To further examine the appearance of germ cell-like cells, various germ cell markers were detected, including one of the earliest PGC markers (IFITM3), early PGC markers (NANOS3 and EPCAM), a late PGC marker (DDX4), a meiosis-specific marker (SYCP3), an oocyte marker (ZP3), and PGC and pluripotency markers (OCT4 and NANOG).^5^^,^^25^^,^^33^ FCM analysis showed that cells positive for IFITM3 and/or EPCAM were detected (Figure 1C; Table S1). The immunofluorescence assay further indicated that germ cell-like cells at different developmental stages, from PGC-like cells to oocyte-like cells, were generated in HL7721 cultures (Figures 1E and S1B). These findings lend support to the presence of PGC-like cells appearing in HL7721 cultures.

As PGCs migrate to the genital ridge and mature, they erase all parental imprints genome-wide.^34^^,^^35^ Consequently, the *H19* differentially methylated region (DMR), a classic imprint-control element established during gametogenesis, must be fully demethylated in gonadal PGCs. To further evaluate the resemblance of PGC-like cells to natural PGCs, we examined whether imprint erasure at DMRs occurred in HL7721 cultures. Strikingly, methylation analysis revealed a fully demethylated DMR state in a subpopulation of HL7721 cells, consistent with the characteristics of a PGC-like state in HL7721 cells (Figure 1F; Table S14).

### PGC-like cells link to tumor initiation

Consistent with TNAP staining results, the PGC-like cells were mainly enriched in the upper layer of HL7721 cultures (Figures 1D and S1A). We then collected the upper layer and bottom layer of HL7721 cells, respectively, by shaking the flask rigorously. FCM analysis showed that the ratio of PGC-like cells in the upper layer was far higher than that in the bottom layer (Figure 1G; Table S2). RT-qPCR analysis showed that the expression levels of a series of germ cell markers, such as *OCT4*, *SOX2*, *NANOG*, *STELLAR*, *NANOS3*, *IFITM3*, *SOX17*, *PRDM1*, *TFAP2C*, and *SYCP3*, were far higher in upper layer cells than in bottom layer cells in HL7721 cultures (Figure 1H; Table S3). To investigate the potential roles of the PGC-like state in tumor initiation of HL7721 cultures, upper-layer and bottom-layer cells from HL7721 (about 1,000 cells/mouse) were injected subcutaneously into nude mice, respectively. Interestingly, the upper-layer subpopulation gave rise to tumors quickly, while the bottom-layer subpopulation barely developed tumors, and about half of the mice injected with bottom-layer cells failed to form tumors within 5 months after injection (Figure 1I). These findings indicated that the PGC-like cells may be closely associated with the tumorigenicity of HL7721 cultures.

Notably, the tumors derived from HL7721 cultures showed the traits of teratocarcinomas in pathology and were positive for OCT4 and SOX2 (Figure 1J). It has been proposed that teratocarcinomas experimentally arise from PGCs,^4^ lending further support to the phenotypic similarities between the PGC-like cells and the natural PGCs. Together, our findings suggest that PGC-like cells are present in HL7721 cultures and may be linked to tumor-initiating ability.

### Requirement of PGC fate-maintaining genes for tumorigenicity

**Figure 2:**
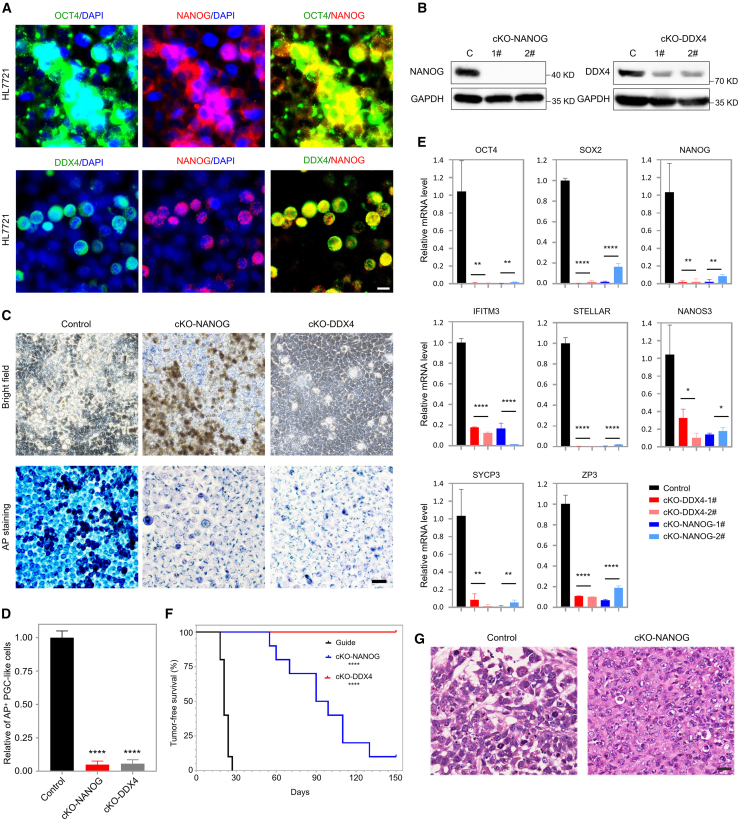
Activation of PGC-like fate is closely linked to tumor initiation in HL7721 cells (A) Immunofluorescence analysis showing the expression of OCT4, NANOG, and DDX4 in the HL7721 cultures. Scale bars, 25 μm. (B) Western blot analysis of NANOG and DDX4 expression in the HL7721 control (guide-only) and respective gene knockout (cKO-*NANOG* and cKO-*DDX4*) groups. (C) Bright-field images showed AP staining in the HL7721 control (guide-only), cKO-*NANOG*, and cKO-*DDX4* cultures, respectively. Scale bars, 25 μm. (D) Statistical analysis of AP-positive cell formation efficiency in the HL7721 control group versus the cKO-*NANOG* and cKO-*DDX4* groups (*n* = 6). Data are presented as mean ± SD and analyzed by unpaired *t* test. (E) RT-qPCR analysis showing differences in the expression of germ cell-related genes in the HL7721 control (guide-only), cKO-*NANOG*, and cKO-*DDX4* groups (*n* = 3). Data are presented as mean ± SD and analyzed by unpaired *t* test. (F) Tumor-free survival curves evaluating the tumor initiation potential of the HL7721 control group (guide-only) versus the cKO-*NANOG* and cKO-*DDX4* groups (*n* = 10). Data are analyzed by log rank test. (G) Representative images of H&E staining in xenograft tumor tissue sections derived from mice in the HL7721 control and HL7721-KO-*NANOG* groups. Scale bars, 20 μm. ∗*p* < 0.05, ∗∗*p* < 0.01, ∗∗∗*p* < 0.001, ∗∗∗∗*p* < 0.0001; n. s., no significant statistical difference.

We further investigated whether the PGC-like state is associated with tumor-initiating ability. In mouse models and hPGC development studies, NANOG and DDX4 are essential for maintaining the PGC fate.^27^^,^^33^ Consistent with the appearance of PGC-like cells, the expression of these two proteins could be detected along with the expression of other germ cell markers in HL7721 cultures (Figure 2A). We then knocked out PGC fate-maintaining-related genes, *NANOG* and *DDX4*, respectively, using CRISPR-Cas9 technology in HL7721 cells (Figure 2B; Table S13). Interestingly, the PGC-like cell clusters failed to survive in response to *NANOG* or *DDX4* knockout (Figure 2C), consistent with the function of these two genes in maintaining PGC fate.^5^ Apoptotic cells were observed frequently in the upper layer of HL7721-KO-*NANOG* cultures (Figure 2C), in agreement with PGC apoptosis found in *NANOG* knockout mice.^5^ The TNAP staining showed that the ability to form PGC-like cells was abruptly inhibited in the knockdown group compared to the control HL7721 group (Figures 2C and 2D; Table S4). Consistently, RT-qPCR analysis showed that the expression of embryonic/germ cell-related genes, including *IFITM3*, *STELLAR*, *NANOS3*, *SYCP3*, *ZP3*, *OCT4*, *SOX2*, and *NANOG*, decreased abruptly in HL7721 cultures following the deletion of *NANOG* or *DDX4* (Figure 2E; Table S5). These findings indicated that these two germ-cell-fate-maintaining genes participate in the regulation of PGC-like cell formation.

We then injected the knockout cells into nude mice to investigate whether the tumorigenicity was also affected. Not surprisingly, the deletion of *NANOG* or *DDX4* markedly impaired tumor initiation in HL7721 cells (Figure 2F). HL7721-KO-*DDX4* cells did not develop tumors within 5 months, whereas the control group formed tumors within 3 weeks (Figure 2F). Notably, xenograft tumor tissues derived from HL7721-KO-*NANOG* showed higher differentiation than those from HL7721 in pathological morphology (Figure 2G). Surprisingly, *DDX4* knockdown suppressed tumorigenicity more robustly than *NANOG*, *SOX17*, or *PRDM1* knockdown in HL7721 cells, indicating that the PGC-like cells might not acquire tumorigenic potential until *DDX4* is activated. Among these, the relevant experimental results after *PRDM1* and *SOX17* knockdown are shown in the eighth section of the Results. On the other hand, we speculate it is possible that, against the complex mutational background of cancer cells, other genes or pathways can at least partially compensate for *NANOG*, *SOX17*, or *PRDM1* loss, whereas such redundancy may not be available for *DDX4*, potentially explaining why its deletion more strongly impairs tumorigenic potential. Collectively, these findings further suggest that the PGC-like cells resemble the natural PGCs in cell fate maintenance, and the ability to form PGC-like cells is likely closely linked to tumor initiation and the maintenance of the poorly differentiated state of hepatic tumor cells.

### Distinct subpopulation cells in HL7721 cells

**Figure 3:**
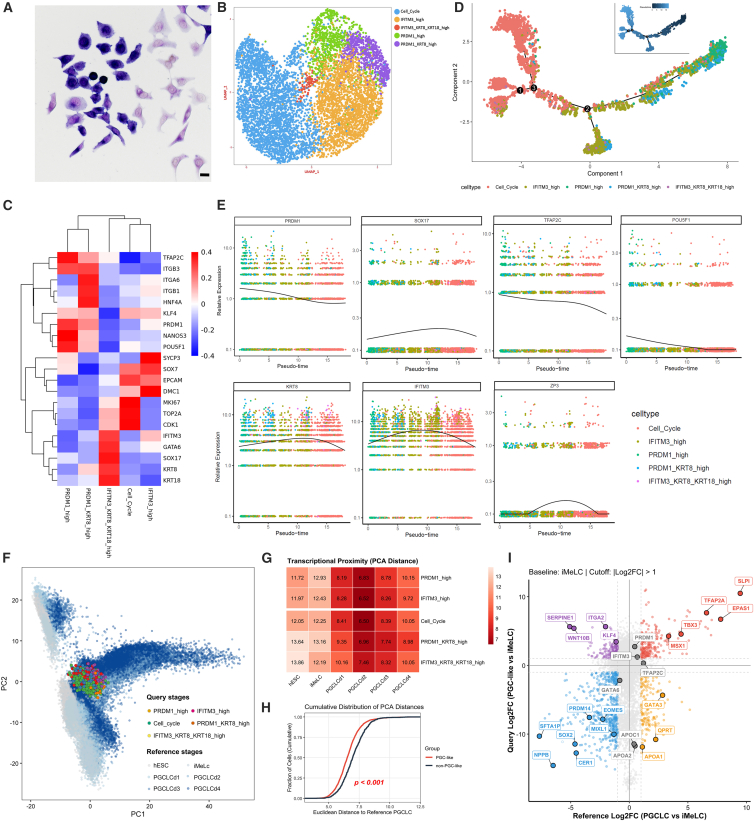
Characterization of distinct cell subpopulations and potential PGC-like specification in HL7721 cultures via single-cell analysis (A) Representative AP staining images of HL7721 cells, indicating subpopulations with distinct morphologies and staining densities. Scale bars, 25 μm. (B) UMAP visualization of single-cell clustering into five distinct subpopulations, including Cell_Cycle, IFITM3_high, IFITM3_KRT8_KRT18_high, PRDM1_high, and PRDM1_KRT8_high clusters. (C) Heatmap of gene expression of key germ cell-related genes (early and late meiosis), and of pluripotency and somatic markers. (D) Pseudotime gradient across the developmental trajectory of HL7721 and overlay of cell subpopulations on this pseudotime trajectory. (E) Dynamic expression profiles of PGC-related genes across pseudotime. (F) PCA projection analysis of HL7721 cells onto the hESC-hPGCLC developmental trajectory. In the plot, colored scatter points represent HL7721 query cells of different subpopulations, while gray and blue scatter points represent the reference developmental stages from hESC induction to hPGCLC day 4. (G) Heatmap quantifying the mean Euclidean distance between HL7721 subpopulations and reference developmental stages in PCA space. Lower values (dark red) indicate higher transcriptional similarity. (H) Cumulative distribution function of Euclidean distances to the reference hPGCLC centroid. The PGC-like group is significantly closer to the reference than the non-PGC-like group (*p* < 0.001, permutation test). (I) Quadrant correlation analysis of DEG Log2FC: HL7721 PGC-like vs. reference hPGCLC (baseline: iMeLC).

The origin of the PGC-like cells remains unclear. Are they from ES-like cells, somatic cells, or cell division of resident PGC-like cells in HL7721 cultures? ES-like cell clones appeared unlikely, as they were not observed in HL7721 cultures. If PGC-like cells originate from somatic cells, the intermediate state resembling PGC fate specification should appear in HL7721 cultures. We next determined whether the PGC-like cells originate from somatic cells. To this end, we investigated distinct subpopulations of cells and their relationship with the PGC-like cell state in HL7721 cultures. The results of TNAP staining and morphology showed that distinct differentiated cells were present in HL7721 cultures, including somatic cells negative for TNAP, PGC-like cells with strong positive TNAP signal and round morphology, and intermediate-state cells (Figure 3A).

Single-cell RNA sequencing (scRNA-seq) was then performed to further understand the distinct cell subpopulations and the formation of PGC-like cells in HL7721 cultures. scRNA-seq revealed that a subset of HL7721 cells expressed a series of genes linked to pluripotency (e.g., *OCT4* and *LIN28A*) and/or PGC specification (e.g., *PRDM1*, *SOX17*, *TFAP2C*, *IFTIM3*, and *NANOS3*) (Figures S2A–S2F). In addition, scRNA-seq showed that distinct cell populations, including hepatic tumor cells, PGC-like tumor cells, late germ cell-like tumor cells, and cells at the intermediate state, existed in the cultures, indicating that the PGC-like fate was activated in the hepatic tumor cells (Figures S2A–S2F). During PGC specification, *PRDM1* is expressed early, followed by the expression of *IFITM3*, one of the earliest markers of PGCs,^33^ whereas *KRT8/KRT18* are markers of hepatic cells. We therefore used these markers to characterize different cell subpopulations. The total cell population was divided into 5 subpopulations via unsupervised t-SNE (t-distributed stochastic neighbor embedding) analysis, including PRDM1_high cells, PRDM1_KRT8_high cells, IFITM3_KRT8_KRT18_high cells, IFITM3_high cells, and cells in the cell cycle, as segregated by lineage markers (Figures 3B and 3C). *PRDM1*, a key regulator of PGC specification, was activated in some tumor cells (Figure S2B). Consistent with the role of *PRDM1* in suppressing the expression of somatic genes during PGC specification, genes related to somatic cells (such as *GATA6*) were expressed at a lower level while genes related to PGC fate (such as *OCT4*, *TFAP2C*, *ITGB3*, and *NANOS3*) were expressed at a higher level in PRDM1_high and PRDM1_KRT8_high cells compared to other groups (Figure 3C). IFITM3_high cells and IFITM3_KRT8_KRT18_high cells may represent a distinct cell fate of early germ cells (Figure 3C). Consistent with this notion, meiosis-related genes *SYCP3* and *DMC1* were highly expressed in the IFITM3_high cells while the expression level of differentiation-related genes such as *GATA6* was higher in the IFITM3_KRT8_KRT18_high cells (Figure 3C). According to the gene expression, the PRDM1_high cells and PRDM1_KRT8_high cells possibly represent the early stage of PGC-like fate activation; IFITM3_high cells possibly represent the late germ cell-like cells; IFITM3_KRT8_KRT18_high cells likely include somatic cells and cells at the intermediate stage, while cell cycle cells possibly include cells at the stage of mitosis and meiosis. These findings indicate that high heterogeneity exists among tumor cells at distinct developmental stages, ranging from somatic cells and intermediate-stage cells to PGC-like cells, in HL7721 cultures.

### Single-cell snapshots capture PGC-like specification

To computationally model the developmental relationships between these distinct cell subpopulations, we performed pseudotime trajectory analysis using Monocle 2. The main trunk of this trajectory appears to originate from cell subpopulations that have already activated the key regulatory factor PRDM1 (including PRDM1_high cells and PRDM1_KRT8_high cells), subsequently differentiating into a more mature PGC-like state (IFITM3_high cells) before entering the SPLC cycle (Figures 3D, S4A, and S4B). Notably, the IFITM3_KRT8_KRT18_high population, which co-expresses somatic markers, was resolved onto a distinct side branch, suggesting that it may represent an alternative cell fate distinct from the primary SPLC path (Figures 3D, S4A, and S4B). Plotting key gene expression along this main trajectory revealed an ordered and dynamic regulatory cascade highly consistent with our SPLC hypothesis (Figure 3E). At the trajectory’s onset, key inducers, including PRDM1, OCT4, SOX17, and TFAP2C, were already active (Figure 3E). As pseudotime advanced, the earliest PGC marker IFITM3 and the oocyte marker ZP3 increased sharply (Figure 3E). Collectively, these analyses support a stepwise acquisition of a PGC-like fate from a PRDM1-activated cell state in HL7721 cultures.

To quantitatively evaluate the similarity of these subpopulations to the true primitive germ cells, we conducted an integrated analysis, comparing our scRNA-seq data with the public reference dataset of hPGC-like cells (hPGCLCs) derived from the highly active UCLA2 human ES cell line. Integrated analysis revealed that the PRDM1+ and IFITM3+ cell clusters in HL7721 cells were closely adjacent to the reference hPGCLCs and exhibited a continuous distribution in the uniform manifold approximation and projection (UMAP) space (Figures S3A–S3D). To further assess the similarity, we performed principal-component analysis (PCA) space integration of HL7721 scRNA-seq data with the reference hPGCLC lineage.^36^ We projected HL7721 cells onto the PCA space of the reference dataset (Figure 3F; Table S15) and calculated the Euclidean distances (Figure 3G; Table S16). The results showed that the PGC-like subclusters (PRDM1_high and IFITM3_high cells) exhibited the highest similarity to hPGCLC d2 with a distance of approximately 6.5–6.8 (Figure 3G). A permutation test confirmed that this transcriptomic proximity was statistically significant (*p* < 0.001, Figure 3H). This comparative analysis provides strong evidence that the SPLC process in HL7721 cells generates a cell state with a transcriptomic profile similar to that of bona fide hPGCLCs, thereby further confirming the existence of the PGC-like state.

Quadrant correlation analysis of differentially expressed genes (DEGs), using induced primordial germ cell-like cells (iMeLCs) as the baseline, revealed that HL7721-derived PGC-like cells significantly recapitulated the gene expression dynamics of hPGCLC^36^^,^^37^ (Figure 3I; Table S17). Specifically, both cell types showed synergistic upregulation of core germline transcription factors, including *PRDM1*, *TFAP2A/C*, and *IFITM3*^36^ (quadrant I), along with the concomitant downregulation of mesendodermal lineage genes such as *MIXL1*, *EOMES*, and *CER1*^38^ (quadrant III). These results indicate that the reprogramming process successfully reconstructed the core molecular framework of germline development and effectively silenced somatic programs. Furthermore, the quadrant analysis identified molecular signatures unique to tumor-derived hPGCLCs (Figure 3I). On the one hand, certain molecules associated with tumor invasion (e.g., *WNT10B* and *SERPINE1*)^39^ remained highly expressed due to the host cell background. On the other hand, the significant suppression of liver-specific metabolic genes (*APOA1/2* and *APOC1*)^40^ demonstrates that the reprogramming process effectively initiated a “lineage clearance” mechanism, erasing the original hepatic identity of HL7721 cells and successfully redirecting their fate toward the germline trajectory. In conclusion, these results provide effective evidence that the SPLC program steers tumor cells toward a PGC-like state.

### Co-activation of key PGC-associated genes in a single cell

**Figure 4:**
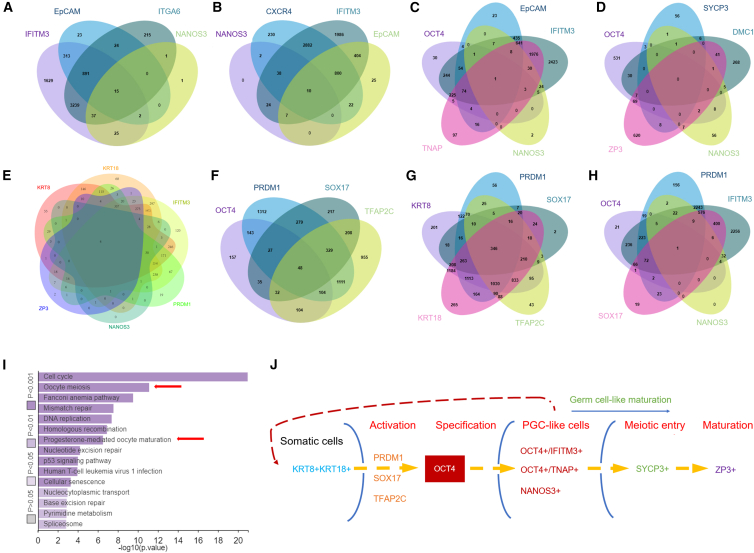
Single-cell co-expression analysis of key PGC-associated genes (A–H) Venn diagrams illustrating the co-expression of key PGC-related genes in HL7721 cultures. (I) Kyoto Encyclopedia of Genes and Genomes (KEGG) pathway enrichment analysis of the Cell_Cycle cell subpopulation. (J) Schematic model of SPLC according to documents and the results of scRNA-seq.

To further investigate the potential specification of a PGC-like identity, we systematically examined the co-expression of key PGC-associated genes. Interestingly, germ cell-related genes at different developmental stages were simultaneously expressed in some cells. A subset of cells highly expressed early PGC-related genes (e.g., *IFITM3*, *NANOS3*, *EPCAM*, and *ITGA6*), meiosis-related genes (*SYCP3* and *DMC1*), or oocyte-related genes (*ZP3* and *GDF9*), but later PGC-related genes (*DDX4*, *DND1*, and *DAZL*) were expressed at very low levels (Figures 4A–4H and S2A–S2F), thus indicating that a subpopulation of cells maintained a germ cell-like state. *EPCAM* can be used as a marker for PGC-like cells derived from induced pluripotent stem cells (iPSCs).^25^^,^^31^ scRNA-seq revealed that subpopulation cells in HL7721 cultures expressed genes related to PGC-like cells at different stages, as suggested by being double-positive for *IFITM3* and *EPCAM*, *IFITM3* and *NANOS3*, *EPCAM* and *NANOS3*, and *EPCAM* and *ITGA6* and/or by co-expressing these four genes (Figure 4A). Together, these findings suggest that SPLC appears in HL7721 cultures.

It has been documented that *SOX17*, *TFAP2C*, and *PRDM1* are essential for hPGC specification.^25^^,^^30^^,^^31^ scRNA-seq revealed that a substantial number of cells in subpopulations expressing *SOX17* (∼14%), *TFAP2C* (∼37%), or *PRDM1* (∼28%) were detected (Figure S2F). Of note, co-activation of *SOX17* and *PRDM1* can induce PGC-like fate in iPSCs and even somatic cells.^31^ scRNA-seq showed that an overlap between *SOX17* and *PRDM1* was detected, supporting activation of PGC-like fate in HL7721 cultures (Figures 4F–4H). Consistent with the notion that *OCT4* partners with *SOX17* to drive the PGC fate,^25^^,^^31^ co-expression of *OCT4* and *SOX17* was also observed in HL7721 cultures (Figures 4F and 4H). Moreover, a series of potential intermediate stages were observed in HL7721 cultures (Figures 4A–4H), and oocyte development-related genes were highly expressed in the cell cycle group (Figure 4I). Based on previous studies^25^^,^^27^^,^^31^^,^^33^^,^^41^ and scRNA-seq results, we modeled a potential pseudotime trajectory involving key sequential events of SPLC in HL7721 cultures (Figure 4J). Collectively, these findings suggest that acquisition of PGC-like cell fate from somatic cells likely occurs in HL7721 cultures.

### Lineage tracing of SPLC

**Figure 5:**
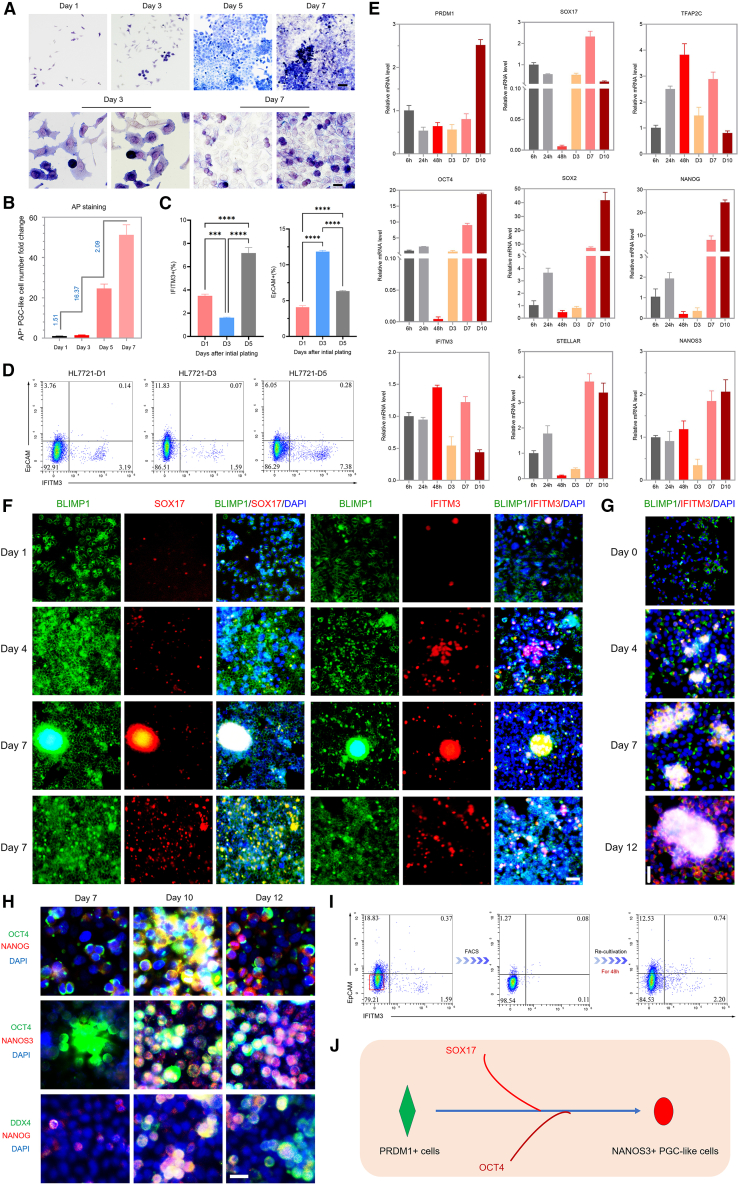
Generation of PGC-like cells from somatic cells (A) Representative bright-field images of AP staining in HL7721 cultures at indicated time points (day 1, day 3, day 5, and day 7) after subculture. Scale bars, 25 μm. (B) Changes of AP^+^ PGC-like cell number with the culture time extension (*n* = 6). (C and D) FCM analysis showing the dynamic ratios of IFITM3^high^ cells and EPCAM^high^ cells with the culture time extension (*n* = 3). Data are presented as mean ± SD and analyzed by one-way ANOVA followed by Tukey’s multiple comparison test. (E) RT-qPCR analysis of the relative expression of germ cell-related genes at different time points after subculture (*n* = 3). Data are presented as mean ± SD. (F–H) Immunofluorescence analysis showing the expression of PGC-related proteins (BLIMP1, SOX17, IFITM3, OCT4, NANOG, NANOS3, and DDX4) at different time points (day 0, day 1, day 4, day 7, day 10, and day 12). Scale bars, 50 (F and G) and 25 μm (H). (I) Emergence of PGC-like cells and SPLC re-establishment from the IFITM3^low^EPCAM^low^ subpopulation *in vitro*. (J) Model depicting the expression of genes related to PGC specification and development in HL7721 cultures as culture time extends after subculture. ∗*p* < 0.05, ∗∗*p* < 0.01, ∗∗∗*p* < 0.001, ∗∗∗∗*p* < 0.0001; n. s., no significant statistical difference.

To monitor the key events driving the acquisition of PGC-like fate, we investigated the sequential activation of PGC fate-related genes at different time points of culture. We analyzed TNAP^high^ PGC-like cell formation at different time points in HL7721 cultures. Some TNAP^high^ PGC-like cells were observed in division (Figure S5A). At day 3 after subculture at low density, orphan PGC-like cells were frequently observed and often attached to other cells, possibly representing nascent PGC-like cells derived from somatic cells (Figures 5A and S5A). The number of TNAP^high^ PGC-like cells decreased initially but increased drastically from day 3 to day 5 (Figures 5A and 5B; Table S6). This significant increase might be attributed to both the proliferation of PGC-like cells and SPLC from day 3 to day 5 (Figures 4B and 5A; Table S6). Of note, FCM analysis showed that the ratio of IFITM3^high^ cells, which might be linked to nascent PGC-like cells, decreased at the beginning from day 1 to day 3 after subculture at low cell density and thereafter increased significantly from day 3 to day 5 (Figures 5C, 5D, and S5B; Table S7), further indicating that the increase was possibly attributed to the formation of nascent PGC-like cells. To further address the derivation of PGC-like cells from somatic cells in HL7721 cultures, we isolated single cells and assessed the ratio of PGC-like cell formation at the single-cell level. Results of single-cell cloning and TNAP staining showed that approximately 75% of single-cell clones could generate PGC-like cells within 3 weeks (Table S8). Together, these findings suggest that some PGC-like cells are spontaneously generated from somatic cells in HL7721 cultures.

Are PGC-like cells derived from division or somatic cells? The key distinction is whether the PGC-like founders originate from somatic cells through PGC-like specification. To further investigate whether PGC-like founders are derived from somatic cells, we therefore investigated whether PGC-like specification occurs prior to the formation of some PGC-like cells. We then performed RT-qPCR to investigate whether a series of PGC specification or germ cell-related genes change with extended culture time after subculture at low density. Overall, the level of *PRDM1* mRNA was maintained at a high level and did not decrease significantly; however, most PGC-specific or fate-maintaining genes decreased initially (Figure 5E; Table S9). The level of *SOX17* decreased drastically and was almost undetectable at 48 h after subculture at low cell density and thereafter increased abruptly (Figure 5E; Table S9). The expression of *SOX17* and *OCT4* increased earlier than that of *IFITM3*, *NANOG*, *NANOS3*, *STELLAR*, and *SOX2* (Figure 5E; Table S9). These findings indicated that PGC specification-activating genes express prior to the formation of new PGC-like cells.

During the course of PGC specification, both BLIMP1 and SOX17 play a crucial role.^31^^,^^41^ We then analyzed changes in the expression of these two proteins with extended culture time. At the early stage, BLIMP1 could be detected in many cells, whereas only a few cells were positive for SOX17 (Figure 5F). With extended culture, the number of BLIMP1- and SOX17-positive cells increased, accompanied by the detection of some double-positive cells (Figure 5F). A series of stages, from low SOX17 expression to strong SOX17 positivity, could be observed in the cultures (Figure 5F). However, this is inconsistent with the notion that *SOX17* induces *PRDM1* expression during the PGC-like specification from iPSCs.^31^

IFITM3-positive cells, which are identified by one of the earliest markers of PGCs,^27^ were barely detected initially but gradually increased and then formed cell clusters (Figures 5F, 5G, and S5C). A series of cells, ranging from IFITM3^−^PRDM1^+^ cells and IFITM3^+^PRDM1^+^ cells to IFITM3^+^PRDM1^−^ cells, were observed with extended culture (Figures 5F and 5G). Cells positive for NANOS3, a specific marker of early PGCs, appeared at the later stage of subculture (Figure 5H). At day 7 after subculture, NANOG- or NANOS3-positive cells were rarely observed when cultured at low density. Consistent with PGC specification, NANOG- or NANOS3-positive cells were easily detected in putative PGC-like cells at day 3 (total day 10) after subculture at high density with upper cells (Figure 5H), supporting that SPLC is activated prior to the activation of hPGC fate in HL7721 cells. Consistent with DDX4 being a late marker of PGCs, DDX4-positive cells were not readily detectable until day 10 following low-density subculture (Figure 5H). In essence, the sequential expression of proteins was BLIMP1, SOX17, and OCT4, followed by NANOS3 and DDX4 in HL7721 cultures (Figures 5F–5H, 5J, and S5D). These findings suggest that PGC-like cell formation may occur through PGC-like specification.

To further explore the induction of PGC-like fate, we compared the OCT4 expression dynamics with those observed during PGC induction from pluripotent stem cells.^42^^,^^43^ OCT4 is both the driver of PGC specification and the guardian of pluripotency, yet its own trajectory is exquisitely stage and site specific. In the early embryo, it shuttles from the cytoplasm (2- to 4-cell stage) to nucleus (morula), reappears in the cytoplasm at the onset of PGC specification,^42^ re-enters the nucleus, vanishes, and finally returns to the cytoplasm in oocytes. In female germ cells, OCT4 is silenced as cells enter meiotic prophase I and is then re-expressed after birth.^43^ We observed a similar choreography when somatic HL7721 hepatocytes were converted to PGC-like cells: OCT4 was first cytoplasmic, then nuclear, and eventually lost in a subset of SYCP3-positive cells. The same sequence—cytoplasmic OCT4 followed by nuclear accumulation and the emergence of SYCP3^+^OCT4^−^ late-stage PGCs—has been reported repeatedly during pluripotent stem cell-to-PGC differentiation.^43^ The parallel dynamics suggest that our induction protocol faithfully reproduces the physiological OCT4 shuttling program.

To further evaluate the existence of SPLC, we isolated the IFITM3^low^EPCAM^low^ double-negative subpopulation by fluorescence-activated cell sorting (FACS), re-cultured these cells, and monitored the emergence of PGC-like cells by FCM. Within 2 days, the double-negative subset generated IFITM3^high^/EPCAM^high^ progeny (Figure 5I). This transition provides further evidence that SPLC can be re-established *in vitro*.

### Potential role of PRDM1 and SOX17 in SPLC and tumorigenicity

**Figure 6:**
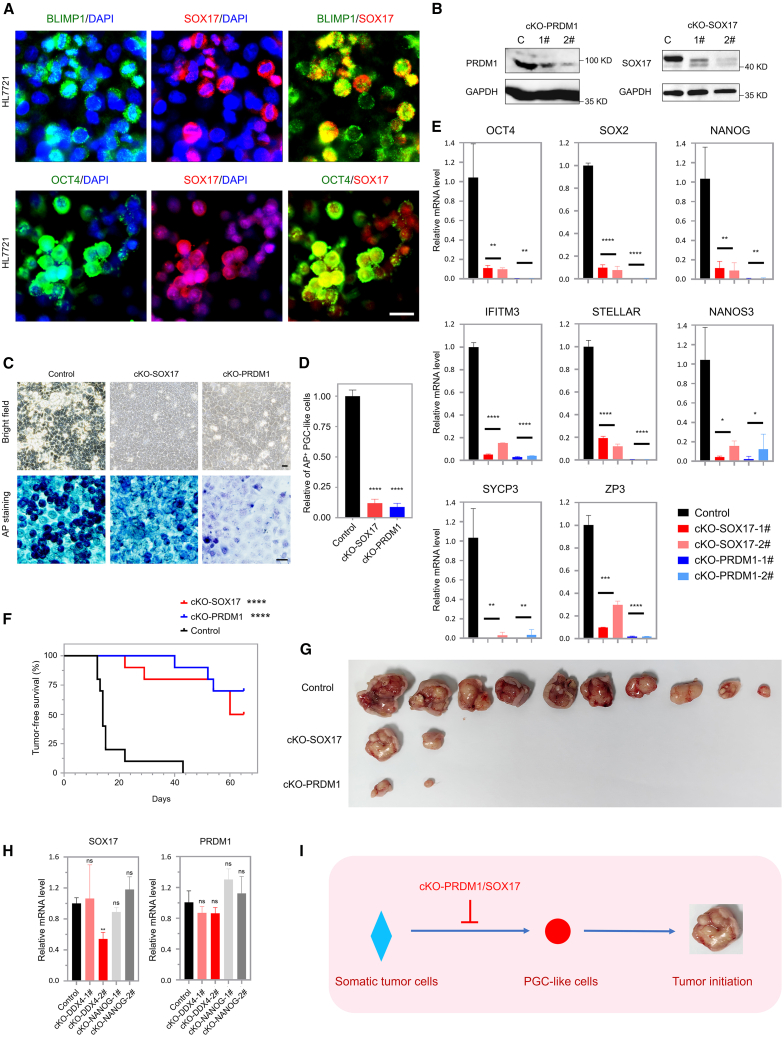
Potential roles of the core genes related to PGC specification in SPLC and tumor initiation of HL7721 cells (A) Immunofluorescence analysis showing the expression of BLIMP1, SOX17, and OCT4 in the HL7721 cultures. Scale bars, 25 μm. (B) Western blot analysis of BLIMP1 and SOX17 expression in the HL7721 control (guide-only) and respective gene knockout (cKO-*PRDM1* and cKO-*SOX17*) groups. (C) Representative bright-field images of AP staining in the HL7721 control (guide-only), cKO-*PRDM1*, and cKO-*SOX17* cultures, respectively. Scale bars, 25 μm. (D) Statistical analysis of AP-positive cell formation efficiency in the HL7721 control group (guide-only) versus the cKO-*PRDM1* and cKO-*SOX17* groups (*n* = 6). Data are presented as mean ± SD and analyzed by unpaired *t* test. (E) RT-qPCR analysis showing differences in the expression of germ cell-related genes in the HL7721 control (guide-only), cKO-*PRDM1*, and cKO-*SOX17* groups (*n* = 3). Data are presented as mean ± SD and analyzed by unpaired *t* test. (F) Tumor-free survival curves evaluating the tumor initiation potential of the HL7721 control group (guide-only) versus the cKO-*PRDM1* and cKO-*SOX17* groups (*n* = 10). Data are analyzed by log rank test. (G) Representative images of tumors derived from mice injected with HL7721 control, cKO-*PRDM1*, or cKO-*SOX17* cells, harvested at 50 days post-injection. (H) RT-qPCR analysis of *PRDM1* and *SOX17* mRNA levels in the HL7721 control, cKO-*NANOG*, and cKO-*DDX4* groups (*n* = 3). (I) Model depicting the potential involvement of PRDM1 and SOX17 in regulating PGC-like cell formation and tumor initiation in HL7721 cultures. ∗*p* < 0.05, ∗∗*p* < 0.01, ∗∗∗*p* < 0.001, ∗∗∗∗*p* < 0.0001; n. s., no significant statistical difference.

To further investigate the SPLC, we investigated whether the upstream key regulators of hPGC specification play important roles in PGC-like cell generation and tumor-initiating ability in HL7721. It has been documented that *SOX17* (instead of *SOX2*) and *PRDM1* are crucial for activating hPGC fate.^25^^,^^31^ The results of scRNA-seq (Figure 3D) and immunofluorescence assays (Figure 6A) showed that a subpopulation of cells co-expressed *SOX17* and *PRDM1*, suggesting the existence of a founder subpopulation of PGC-like cells. Consistent with the notion that OCT4 partners with *SOX17* to drive the PGC fate,^25^^,^^31^ co-expression of *OCT4* and *SOX17* was observed by scRNA-seq (Figure 3D) and immunofluorescence (Figures 6A and S5D). After knocking down *PRDM1* or *SOX17* using CRISPR-Cas9 technology (Figure 6B; Table S13), the PGC-like cell formation decreased markedly (as measured by alkaline phosphatase [AP] staining) in the *PRDM1-*knockdown or *SOX17-*knockdown groups compared with control HL7721 cells (Figures 6C and 6D; Table S4). Consistent with this, the expression of a series of genes was suppressed significantly, such as *NANOS3*, *IFITM3*, *STELLAR*, *NANOG*, *SOX2*, *SYCP3*, and *ZP3* (Figure 6E; Table S5). We then injected HL7721 cells with *PRDM1* or *SOX17* knocked down, as well as control cells, into nude mice, respectively. The results of animal experiments showed that the tumor-initiating ability in HL7721 cultures with *PRDM1* or *SOX17* knocked down declined significantly compared with that in the control group (Figures 6F and 6G).

As aforementioned, KO-*NANOG* or KO-*DDX4* significantly suppressed PGC-like cell survival and tumorigenicity (Figures 2C–2F); however, the expression of *PRDM1* and *SOX17* did not change significantly in KO-*NANOG* or KO-*DDX4* cultures, unlike the PGC-like cell formation and tumorigenicity (Figure 6H). These observations suggest that the suppression of tumor initiation following KO-*PRDM1* or KO-*SOX17* may involve the inhibition of PGC-like fate activation in HL7721 cultures. Overall, these findings suggest that *PRDM1* and *SOX17* act as important inducers of SPLC in HL7721 cultures and that SPLC conversion is likely closely linked to tumor initiation in HL7721 cells (Figure 6I).

### BMP pathway participates in the regulation of SPLC

**Figure 7:**
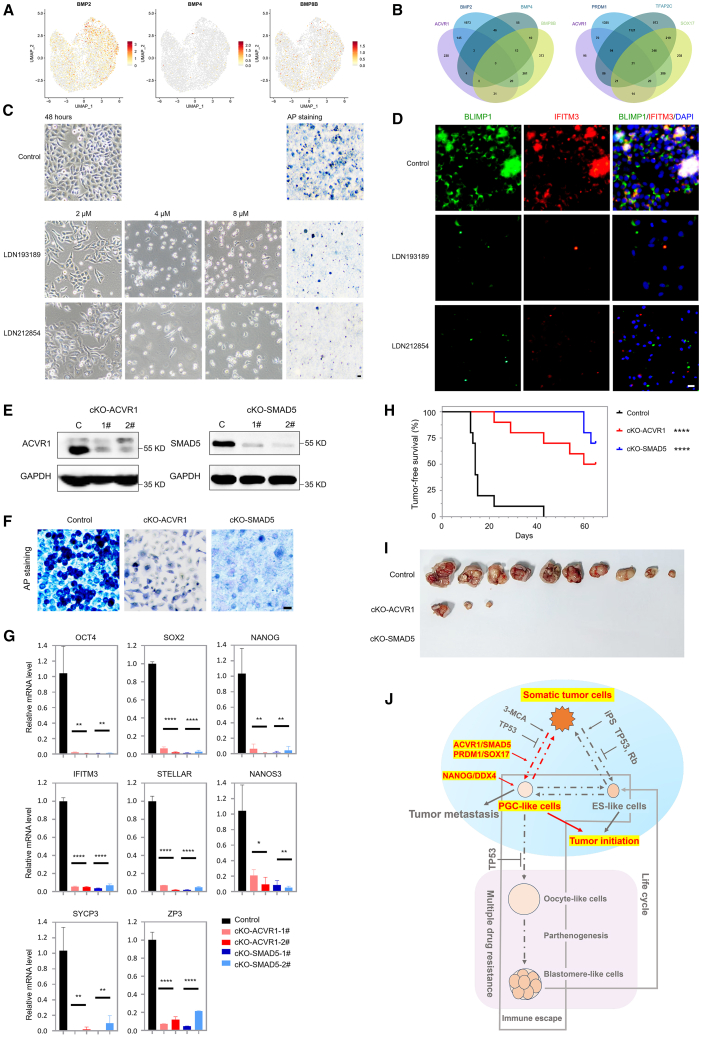
Potential involvement of the BMP pathway in SPLC and tumor initiation of HL7721 cells (A) UMAP visualization of BMP2, BMP4, and BMP8B expression profiles. (B) Venn diagrams illustrating the co-expression of indicated BMP-related genes. (C) Bright-field images of HL7721 cultures treated with DMSO (control), LDN193189, or LDN212854 for 48 h (left), and representative AP staining images in the corresponding cultures (right). Scale bars, 25 μm. (D) Immunofluorescence analysis showing the expression of BLIMP1 and IFITM3 in the HL7721 control and drug-treated (LDN193189/LDN212854) groups. Scale bars, 25 μm. (E) Western blot analysis of ACVR1 and SMAD5 expression in the HL7721 control (guide-only) and respective gene knockout (cKO-*ACVR1* and cKO-*SMAD5*) groups. (F) Representative bright-field images of AP staining in the HL7721 control (guide-only), cKO-*ACVR1*, and cKO-*SMAD5* cultures, respectively. Scale bars, 25 μm. (G) RT-qPCR analysis showing differences in the expression of germ cell-related genes in the HL7721 control (guide-only), cKO-*ACVR1*, and cKO-*SMAD5* groups (*n* = 3). Data are presented as mean ± SD and analyzed by unpaired *t* test. (H) Tumor-free survival curves evaluating the tumor initiation potential of the HL7721 control group (guide-only) versus the cKO-*ACVR1* and cKO-*SMAD5* groups (*n* = 10). Data are analyzed by log rank test. (I) Representative images of tumors derived from mice injected with HL7721 control (guide-only), cKO-*ACVR1*, and cKO-*SMAD5* cells, harvested at 50 days post-injection. (J) Schematic diagram integrating current findings with previous literature and studies to link distinct malignant behaviors. The yellow indicates our current findings. The gray lines represent conclusions derived from our previous studies or documents. ∗*p* < 0.05, ∗∗*p* < 0.01, ∗∗∗*p* < 0.001, ∗∗∗∗*p* < 0.0001; n. s., no significant statistical difference.

To further explore the regulatory mechanism of SPLC, we investigated the signaling pathway of SPLC. The BMP pathway plays essential roles in PGC specification^25^; therefore, we investigated its potential role in the SPLC and tumor initiation. scRNA-seq results showed that a series of genes related to the BMP/SMAD pathway, such as *BMP2*, *BMP4*, and *BMP8B*, were expressed in a subpopulation of HL7721 cells (Figure 7A). Moreover, *ACVR1* (BMP type I receptor) could be detected in some HL7721 cells (Figure 7B). A few ACVR1^high^ cells also expressed *BMP2*, *BMP4*, or *BMP8B*, whereas most ACVR1^high^ cells co-expressed *PRDM1*, *SOX17*, and *TFAP2C* (Figure 7B). Since ACVR1 is essential for PGC specification,^33^ we then treated HL7721 cells with BMP type I receptor-blocking agents LDN193189 and LDN212854, respectively, which inhibit activin receptor-like kinases 2/3. The results showed that HL7721 cell viability was reduced by LDN193189 and LDN212854 in a time- and dose-dependent manner (Figures 7C, S6A, and S6B), indicating that BMP signaling may be involved in the survival of HL7721 cells. We found that LDN193189 and LDN212854 at a low dose repressed the formation of PGC-like cells compared with the vehicle control (Figures 7C, 7D, and S6C; Table S10).

We then investigated whether downstream components of the BMP pathway for PGC specification, *ACVR1* and *SMAD5*,^27^^,^^33^ participate in PGC-like cell formation in HL7721 cultures. After knocking down *ACVR1* or *SMAD5* using CRISPR-Cas9 technology (Figure 7E; Table S13), the PGC-like cell formation decreased markedly, as determined by AP staining (Figures 7F and S6D; Table S4). Consistent with this, the expression of a series of downstream genes, such as *OCT4*, *SOX2*, *NANOG*, *SOX17*, *TFAP2C*, *IFITM3*, *STELLAR*, *NANOS3*, *SYCP3*, and *ZP3*, was suppressed (Figure 7G; Table S5). *In vivo* xenograft tumor assays revealed that the tumor initiation ability in HL7721 cultures with *ACVR1* or *SMAD5* knocked down declined significantly compared with the control group (Figures 7H and 7I). Collectively, our findings suggest a potential link between PGC-like cell formation and tumor initiation, lending support to the idea that SPLC is associated with the BMP signaling pathway and may play an important role in tumor initiation.

## Discussion

Specification of PGCs marks the determination of PGC fate and the beginning of the totipotent state.^33^ Our study suggests that PGC-like specification from somatic cells can be activated and may contribute to tumor initiation in the human hepatic tumor cell line HL7721. In HL7721 cultures, tumor cells at different developmental stages appear, progressing from somatic tumor cells through intermediate stages to the PGC-like stage and late germ cell-like stage, consistent with the activation of SPLC. Mechanistically, the SPLC is regulated by core factors, including the BMP signaling pathway, PRDM1, and SOX17, and plays an important role in tumorigenicity in HL7721 cultures. As a result, knockdown of either PGC specification inducers (*ACVR1*, *SMAD5*, *PRDM1*, or *SOX17*) or PGC fate-maintaining genes (*NANOG* or *DDX4*) suppressed both SPLC and tumor initiation, lending support to the idea that the PGC-like state originates from somatic cells and may be closely linked to the acquisition of tumor-initiating ability. Our findings suggest that the activation of SPLC really exists in the human hepatic tumor cell line HL7721 and represents a potential way to acquire tumor-initiating ability and that the signaling pathways of PGC specification and development may also participate in regulating this conversion. These findings could offer insights into the nature and sequence of molecular events underlying the acquisition of tumor-initiating ability.

Intriguingly, somatic-to-PGC conversion could happen spontaneously in some lower organisms,^15^ which might be an ancient way leading to pluripotency. In mammals, mutual conversion of cell fate between germ cells and somatic cells is rigorously prevented.^26^^,^^33^ However, derivation of PGC-like cells from somatic cells under some genetically modified conditions has been demonstrated in some mammals.^15^^,^^44^ Of note is that the mutation of numerous tumorigenesis-related genes, such as TP53 and pRB, could induce the somatic-to-PGC conversion.^15^ It has been documented that famous cancer testis (CT) antigens are expressed extensively in various tumor types,^6^^,^^7^^,^^24^ and germ cell-like cells appear in a variety of tumor cell types and are induced by *TP53* deletion or chemical carcinogen exposure.^17^^,^^18^^,^^19^^,^^21^^,^^22^^,^^45^^,^^46^ In some clinical cases, it has been demonstrated that some germ cell tumors originate from somatic cells, while some somatic tumors derive from germ cells.^47^^,^^48^ Moreover, our previous studies have reported that SPLC could be activated in the mouse breast cell line 4T1.^44^ In the current study, our results suggest that SPLC could also be activated in human somatic tumor cells. Hence, our findings lend further support to the possibility that SPLC is present in mammal tumors, consistent with the notion that in mammals, the mutual conversion of cell fate between germ cells and somatic cells is rigorously prevented under physiologic conditions but might be activated under pathologic condition, such as carcinogenesis. That is, the ancient way from somatic cells back to PGC-like cells in some lower organisms might be reactivated under tumor progression in mammals, including humans.

Tumor progression is a complicated process that involves numerous key events, such as tumor initiation, metastasis, and drug resistance.^1^ It has been documented that early PGCs have intrinsic abilities to endow tumor initiation, migration (corresponding to metastasis), proliferation, and gametogenesis.^30^ Based on the high similarities between germ cell development and tumor formation, Old et al. proposed that reacquiring the PGC-like fate in somatic cells is a driving force for tumor malignant progression.^23^^,^^24^ Consistent with this notion, it has been documented that certain genes related to PGC fate acquisition or maintenance are crucial for tumor initiation,^6^^,^^7^^,^^8^^,^^10^^,^^12^ and the PGC-like state is reported to be important for mouse tumor initiation and hepatic metastasis.^44^^,^^46^^,^^49^ Our previous studies reported that PGC-like cells could give rise to oocyte-like cells resembling gametogenesis; the latter could generate pathogenic blastomere-like cells (tumor initiation) and then establish an independent life cycle.^17^^,^^21^^,^^22^ Moreover, the oocyte-like cells and blastomere-like cells play important roles in driving multidrug resistance and tumor recurrence.^22^ Interestingly, polyploid giant cancer cells (PGCCs) are characterized by large cell size and multinucleated cells, cause multidrug resistance, give rise to progeny cells, induce tumorigenicity, and have been validated as blastomere-like cells,^50^^,^^51^ indicating that the PGC-like cells could link to the PGCCs of pathologic morphology through the germ cell-like development and parthenogenetic activation. Together, PGC-like tumor cells might be the core of tumor malignant traits, and clarifying the origin of PGC-like tumor cells is critical. Therefore, the observation of SPLC activation and the potential roles of SPLC in tumor initiation in human tumor cells not only provide evidence for the Old’s hypothesis but also further emphasize our concept that the reactivation of the embryonic/germ cell-like developmental axis in tumors links various core tumor malignant behaviors together (Figure 7J),^22^^,^^23^^,^^24^^,^^48^ possibly representing the true “stemness” of tumor malignant progression. It also further indicates that the malignant nature of tumors may be abnormal gametogenesis rather than abnormal growth, which explains why somatic tumors extensively exhibit high germ cell traits.^6^^,^^7^^,^^24^

Of note, PGCs are the core of the embryonic/germ cell-like developmental axis in tumors (Figure 7J), which consists of a sequence: somatic cells to PGC-like tumor cells (tumor initiation and metastasis) to oocyte-like cells (drug resistance) to blastomere-like cells (drug resistance and tumor initiation) to somatic cells. Therefore, targeting SPLC might be a potential strategy to prevent tumor malignant progression. Our findings indicate that the SPLC is likely regulated by key genes involved in hPGC specification and fate maintenance.^33^ This is supported by the observation that knocking down PGC-related genes could repress the SPLC and tumor initiation and that the ACVR1 inhibitor could suppress PGC-like cell formation and ultimately kill tumor cells when used at a higher concentration. Unlike mouse PGC specification, hPGC specification is driven by SOX17 rather than SOX2 as a core factor. Consistent with this, knocking down SOX17 suppresses both SPLC and tumor initiation in human hepatic HL7721 tumor cultures. In light of our findings, targeting the pathway or key genes that maintain PGC fate could be a potential strategy for targeting human tumor progression.

In summary, our findings provide insights into SPLC lineage tracing and the origin of tumor initiation in human tumor cells, suggesting a potential step in tumor evolution. Our findings elucidate a possible origin of tumor-initiating ability, are consistent with Old’s concept,^23^^,^^24^ and offer potential targets for human tumor therapy.

### Limitations of the study

However, the pathway of SPLC might not be fully identified toward PGC specification, owing to the distinct context and complicated gene mutations during carcinogenesis. Further studies will be required to determine whether the SPLC is common and to identify other core genes regulating the SPLC in malignant human tumor cells. In addition, the present study focuses on the association between SPLC and tumor-initiating ability. Future studies will further explore the association between SPLC and other malignant phenotypes of tumors (such as metastasis and drug resistance), so as to clarify the comprehensive role of SPLC in the overall progression of tumors. In addition, the overall signal of expression patterns of PGC-related genes is relatively weak, which may be attributed to insufficient sequencing depth. Further sequencing is therefore required to fully characterize their expression profiles.

## Resource availability

### Lead contact

For further details and to obtain resources or reagents, please send your requests directly to the lead contact, Chunfang Liu (chunfang_liu@fudan.edu.cn).

### Materials availability

No new and unique reagents were created in the course of this study, and all materials utilized can be obtained commercially.

### Data and code availability

• scRNA-seq data have been deposited in the SRA and GEO databases and are publicly accessible. The accession numbers are provided in the key resources table. The SRA accession is PRJNA1326419 (run accession: SRR35335378), and the GEO accession is GSE330850.
• No original code was developed or used in the course of this research.
• Any additional information required to reanalyze the data reported in this paper is available from the lead contact upon request.

## Acknowledgments

This work was supported by the 10.13039/501100001809National Natural Science Foundation of China (no. 82273113), 10.13039/100007219Shanghai Natural Science Foundation of China (no. 21ZR1410900), Shanghai Municipal Key Clinical Specialty of China (shslczdzk03303) to C.L., and 10.13039/501100001809National Natural Science Foundation of China (no. 82372327) to Z.M.

## Author contributions

J.L., Z.M., and F.Z. performed most of the experiments; A.L. performed cell culture; P.L. performed a part of animal experiments; J.X. performed the pathologic diagnosis; C.L. designed the experiments, analyzed the data, and wrote the manuscript; and H.-K.L. offered advice and critically reviewed the manuscript.

## Declaration of interests

The authors declare no competing interests.

## STAR★Methods

### Key resources table

**Table:** 

REAGENT or RESOURCE	SOURCE	IDENTIFIER
**Antibodies**		

SOX17	Abcam	Cat#ab224637; RRID: AB_2801385
SMAD5	Abcam	Cat#ab40771; RRID: AB_777981
ACVR1	Abcam	Cat#ab155981; RRID: AB_2929006
OCT4	Abcam	Cat#ab19857; RRID: AB_445175
SYCP3	Abcam	Cat#ab15093; RRID: AB_301639
ZP3	Proteintech	Cat#21279-1-AP; RRID: AB_11124319
NANOG	Abcam	Cat#ab21624; RRID: AB_445175
NANOS3	Abcam	Cat#ab70001; RRID:AB_1269517
DDX4	Abcam	Cat#ab27591; RRID: AB_1269517
BLIMP1	Novus Biologicals	Cat#NB600-235; RRID: AB_3193820
IFITM3	Abcam	Cat# ab15592; RRID:AB_2122095
SOX2	Abcam	Cat#ab171380; RRID: AB_2732072
GAPDH	Proteintech	Cat#60004-1-Ig; RRID: AB_2107436
HRP goat anti-rabbit IgG	BIOMIKY	Cat#MK103; RRID: AB_3678811
HRP goat anti-mouse IgG	BIOMIKY	Cat#MK101; RRID: AB_3678810
Alexa Fluor® 488 Anti-Fragilis antibody	Abcam	Cat#ab198559
Alexa Fluor® 647 Anti-EpCAM antibody	Abcam	Cat#ab237396
Goat anti-Rabbit IgG (Heavy chain) Superclonal™ Secondary Antibody, Alexa Fluor™ 555	Thermo Fisher Scientific	Cat# A27039, RRID:AB_2536100
Goat anti-Mouse IgG (H+L) Cross-Adsorbed Secondary Antibody, Alexa Fluor™ 488	Thermo Fisher Scientific	Cat# A11001, RRID:AB_2534069

**Chemicals, peptides, and recombinant proteins**		

LDN-193189	Sigma-Aldrich	Cat#SML0559
LDN-212854	Sigma-Aldrich	Cat#SML0965
Blasticidin	Sigma-Aldrich	Cat#203350
Puromycin	MedChemExpress	Cat#HY-B1743
X-Gal	Biosharp	Cat#BL546A
IPTG	Biosharp	Cat#BL545A
TRIzol	Invitrogen	Cat#15596026CN
X-tremeGENE^TM^ 9 DNA Transfection Reagent	Roche	Cat#06365787001
Polybrene	Sigma-Aldrich	Cat#TR-1003

**Critical commercial assays**		

PrimeScript^TM^ RT Master Mix	Takara	Cat# RR036A
TB Green® Premix Ex Taq^TM^ II	Takara	Cat# RR820A
Alkaline phosphatase (AP) staining kit	Vector Laboratories	Cat# SK-5300
BeyoECL Star kit	Beyotime	Cat# P0018AM
IHC Detect Kit for Rabbit/Mouse Primary Antibody	Proteintech	Cat# PK10006
Quick-DNA^TM^ Miniprep Kit	Zymo Research	Cat# D3024
EZ DNA Methylation-GOLD^TM^ Kit	Zymo Research	Cat# D5005
Zymo *Taq*^TM^ PreMix	Zymo Research	Cat# E2003
E.Z.N.A.® Gel Extraction Kit	Omega	Cat# 2500
pMD^TM^19-T Vector Cloning Kit	Takara	Cat# 6013

**Deposited data**		

scRNA-seq dataset for hPGCLCs	GEO	GEO: GSE140021
scRNA-seq dataset for HL7721	This study	SRA: PRJNA1326419; GEO: GSE330850

**Oligonucleotides**		

sgRNA	Sigma-Aldrich	Table S12

**Recombinant DNA**		

lentiCas9-Blast	Addgene	Plasmid #52962

**Software and algorithms**		

BD Rhapsody Analysis Pipeline (version: 1.9.1)	BD Biosciences	https://www.bdbiosciences.com/en-us/products/reagents/single-cell-analysis/rhapsody-single-cell-analysis-system
bcl2fastq (version: 2.20.0)	Illumina	https://support.illumina.com/sequencing/sequencing_software/bcl2fastq-conversion-software.html
UMI-tools (11.2 version)	Smith et al.^52^	https://umi-tools.readthedocs.io/en/latest/index.html
STAR (version: 2.5.1)	Dobin et al.^53^	https://code.google.com/archive/p/rna-star/
Seurat (version: 5.0.1)	Hao et al.^54^	https://github.com/satijalab/seurat
Monocle 2	Qiu et al.^55^	http://cole-trapnell-lab.github.io/monocle-release
pheatmap	Kolde et al.^56^	https://cran.rstudio.com/
VennDiagram (version: 1.7.1)	Chen et al.^57^	https://github.com/cran/VennDiagram
GSEApy (version: 0.10.1)	Subramanian et al.^58^	https://github.com/zqfang/GSEApy
ggplot2 (version: 3.2.5)	Wickham et al.^59^	https://ggplot2.tidyverse.org/
R (v4.3.2)	N/A	https://www.R-project.org/

### Experimental model and study participant details

#### Cell lines

All cell lines (SMMC7721 and HEK293T) were obtained from the National Collection of Authenticated Cell Cultures, Chinese Academy of Sciences. All cell lines were authenticated by STR profiling and confirmed to be mycoplasma free.

#### Mice

Female BALB/c nude mice (4-6 weeks old) were obtained from Shanghai Model Organisms. All animal protocols were approved by the Animal Ethics Committee of Fudan University (approval number: 2022JS Huashan hospital-075) and conformed to the principal guidelines of the Guide for the Care and Use of Laboratory Animals (8th edition, National Academies Press).

### Method details

#### Cell culture and single-cell cloning

Cells were cultured in Dulbecco’s modified Eagle’s medium (DMEM; Gibco) supplemented with 10% fetal bovine serum (FBS; Sigma-Aldrich), 100 units/ml penicillin, and 100 μg/ml streptomycin (Gibco). Cells were cultured at 37°C in a humidified incubator containing 5% CO_2_. Cells were spread to multi-well plates at the peak of proliferation for expriments.

For single-cell cloning, SMMC7721 cells were plated at 0.5 cells/well in 96-well plates. Wells with a single attached cell were selected and cultured for 2-3 weeks until the formation of individual cell clones were observed.

#### Drug treatment and crystal violet staining

HL7721 cells were plated in six-well culture plates at 1×10^5^ cells per well. The next day, cells were exposed to varying concentrations of LDN193189 (Sigma-Aldrich) and LDN-212854 (Sigma-Aldrich), specifically at 2 μM, 4 μM, and 8 μM. Cell counts and morphological alterations were meticulously documented at different time intervals post-treatment. Subsequently, immunofluorescence, tissue-nonspecific alkaline phosphatase (TNAP) staining, and crystal violet staining were performed. The cells were stained with a 0.1% crystal violet solution in methanol for 20 minutes. After washing three times with double-distilled water, the cells were visualized under a microscope, and representative images were captured.

#### Quantitative real-time PCR (qRT-PCR)

Total RNA was isolated from each culture with TRIzol reagent (Invitrogen). Approximately 1500 ng of total RNA was reverse-transcribed using PrimeScript^TM^ RT Master Mix (Takara) under the following conditions: 37°C for 15 min, followed by 85 °C for 5 s. The resulting cDNA was diluted 1:3 in nuclease-free water before use. Quantitative real-time PCR was carried out in 96-well plates with TB Green® Premix Ex TaqTM II (Takara) on a 7500 Real-Time PCR System (Applied Biosystems). Thermal cycling conditions were: 95 °C for 1 min, followed by 40 cycles of 95°C for 15 s and 60°C for 1 min. A melting-curve stage (95°C 15 s, 60°C 1 min, ramp 0.5°C s^-1^ to 95°C) was appended to verify specificity. Each experiment was performed in triplicate. Gene expression was normalized to GAPDH and quantified by the 2^-ΔΔCt^ method. All primers were designed to span an intron ≥1 kb, ensuring amplification of only the spliced cDNA. Primer sequences are listed in Table S11.

#### Bisulfite sequencing

First, genomic DNA was extracted from HL7721 cells using the Quick-DNA^TM^ Miniprep Kit (Zymo Research). Subsequently, the genomic DNA was treated with the EZ DNA Methylation-GOLD^TM^ Kit (Zymo Research) to convert methylated cytosines into uracils. After that, nested PCR was performed using Zymo Taq^TM^ PreMix (Zymo Research) to amplify the CpG island fragment of the human *H19* DMR region (primer sequences are shown in Table S11). The PCR products were subjected to gel extraction using the E.Z.N.A.® Gel Extraction Kit (Omega), and then ligated into the T-vector with the pMD^TM^19-T Vector Cloning Kit (Takara). The ligation products were transformed and plated, followed by blue-white screening using plates containing X-gal, IPTG, and ampicillin. Finally, single colonies were picked for sequencing. All procedures were carried out in accordance with the manufacturer’s instructions.

#### Single-cell RNA sequencing and analysis

The single-cell suspension of HL7721 was used for single-cell sequencing. After multiple rounds of filtration and viability assessment, the BD Rhapsody system was employed to capture the single-cell transcriptome. The cell suspension was distributed into >200,000 microwells via a limited dilution method, and barcode beads were added to achieve “one bead per cell” pairing. Following cell lysis, mRNA hybridized with the beads. After reverse transcription and ExoI digestion, libraries were prepared using the BD Rhapsody WTA workflow, with cDNA labeled with unique molecular identifiers (UMIs) and cell barcodes. Libraries were quantified using an Agilent High Sensitivity DNA Chip and Qubit Assay, and finally sequenced on an Illumina sequencer with 150 bp paired-end reads.

Subsequently, data analysis was conducted by NovelBio Co., Ltd. using the NovelBrain platform: fastp was used to filter adapter sequences and low-quality reads for clean data; UMI-tools identified the cell barcode whitelist; STAR was applied to map the UMI-based clean data to the human genome GRCh38 for UMI counting. Cells with >200 expressed genes and a mitochondrial UMI rate <20% were retained. Seurat was used for data normalization and to construct PCA, tSNE, and UMAP. Graph-based clustering (resolution = 0.8) was performed, and marker genes were screened under the criteria of log2FC > 0.25, *p* < 0.05, and min.pct1 > 0.1.

Finally, multiple specialized bioinformatics packages were used for subsequent in-depth analysis and plotting. Monocle2 constructed single-cell differentiation trajectories to clarify HL7721 cell subpopulation development paths. GSEApy conducted gene set enrichment analysis to identify significantly active/suppressed biological pathways. Pheatmap generated heatmaps to show marker/differentially expressed gene expression patterns, while VennDiagram created Venn diagrams to compare overlapping/unique gene sets. GSEApy was also used again to validate enrichment results, ensuring analysis reliability.

In addition, to quantitatively assess the similarity between HL7721 subpopulations (query cells) and bona fide hPGCLCs, we performed an integrative analysis with a public scRNA-seq dataset of hPGCLCs derived from the UCLA2 cell line (reference dataset; Chen et al., 2019; GEO accession: GSE140021). The reference dataset and our HL7721 data were integrated using the standard Seurat v5 workflow. This procedure identifies cross-dataset anchors to correct for batch effects, enabling direct comparison of cell states in a shared UMAP embedding. To quantify similarity, query cells were projected onto the PCA space constructed from the reference dataset. Euclidean distances were calculated between query clusters and reference stage centroids using the top 30 PCs. Statistical significance was assessed via a permutation test (10,000 iterations). Quadrant Differential Expression Analysis Log2 Fold Changes (Log2FC) were calculated for “Query PGC-like vs. iMeLC” and “Reference PGCLC vs. iMeLC”. Genes were categorized into quadrants based on Log2FC direction.

#### CRISPR/Cas9-mediated gene knockout

In brief, cells were plated to achieve 60-70% confluency at the time of transfection. The X-tremeGENE^TM^ 9 DNA Tranfection Reagent (Roche) was mixed with Opti-MEM (Gibco) and allowed to incubate for 5 minutes. Then, lentiviral packaging vectors (Beyotime, L00002M) and lentiCas9-Blast (Addgene, Plasmid #52962) were added to the mixture and incubated for 20 minutes. HEK293T cells were transfected with the above mixture. After 6–8 hours, the medium was replaced with DMEM containing 10% FBS. The culture medium supernatant was collected 24 hours after transfection, filtered through a 0.45 μm Steri-Flip filter (Millipore), added to HL7721 cells and cultured for 4 h. To improve transfection efficiency, polybrene (Sigma-Aldrich) was included at a concentration of 7 μg/ml. Repeat transfection twice as described above. Following a 48-hour incubation with the viral supernatant, selection with blasticidin (Sigma-Aldrich) was performed for 3-5 days to obtain single-cell clones, which were further expanded. Subsequently, using the same method, sgRNAs (Sigma-Aldrich, see Table S12) were transfected into HL7721-Cas9 cells, followed by selection with puromycin (MedChemExpress) and isolation of single-cell clones. Gene knockout was confirmed by DNA sequencing (Table S13) and Western blot.

#### Western blotting

The cells were rinsed twice with ice-cold phosphate-buffered saline (PBS), resuspended in radioimmunoprecipitation (RIPA) buffer supplemented with a cocktail of proteinase inhibitors, lysed on ice for 30 minutes, and then centrifuged at 4°C at 15,000 rpm for 20 minutes. The supernatant was collected and boiled for 10 minutes. Lysates were loaded onto a 10% SDS-PAGE gel and transferred onto a polyvinylidene fluoride (PVDF) membrane (0.22 μm, Millipore). Membranes were blocked in 5% low-fat dried milk in Tris-buffered saline with Tween-20 (TBSTw) for 2 hours at room temperature and then incubated with the primary antibodies overnight at 4°C. Primary antibodies included anti-SMAD5 (Abcam, ab40771), anti-SOX17 (Abcam, ab224637), anti-NANOG (Abcam, ab21624), anti-DDX4 (Abcam, ab27591), anti-ACVR1 (Abcam, ab155981), anti-BLIMP1 (Novus Biologicals, NB600-235), and anti-GAPDH (Proteintech, 60004-1-Ig). Membranes were washed and incubated with HRP goat anti-rabbit IgG (BIOMIKY, MK103) and HRP goat anti-mouse IgG (BIOMIKY, MK101) at room temperature for 1 hour. Signal was detected using BeyoECL Star kit (Beyotime).

#### Flow cytometry analysis and fluorescence-activated cell sorting

The cells were incubated with Alexa Fluor® 488 Anti-Fragilis antibody (Abcam, ab198559) and Alexa Fluor® 647 Anti-EpCAM antibody (Abcam, ab237396) on ice for 50 minutes. After washing twice with PBS, the samples were analyzed using a CytoFLEX flow cytometer (Beckman Coulter). To obtain the IFITM3^low^EpCAM^low^ cell population, the cells were processed as described above, filtered through a 40 μm cell strainer, and then subjected to flow sorting using a CytoFLEX SRT flow cytometer (Beckman Coulter).

#### Immunofluorescence

The cells were plated onto chamber slides. After 24 h, cells were fixed in 4% paraformaldehyde at room temperature for 20 minutes and then incubated with primary antibodies overnight at 4°C. After washing, the cells were incubated with secondary antibodies conjugated fluorescent dye, including anti-rabbit Alexa Fluor™ 555 (Thermo Fisher Sci., A27039) and anti-mouse Alexa Fluor™ 488 (Thermo Fisher Sci., A11001). The cell nuclei were visualized by staining with 4′,6-diamidino-2-phenylindole (DAPI; Invitrogen).

#### Tissue-nonspecific alkaline phosphatase (TNAP) staining

The cells were fixed with 4% paraformaldehyde, washed twice with Tris-HCl (pH 8.6), followed by staining with the Alkaline phosphatase (AP) staining kit (Vector Laboratories) in the dark for approximately 45 minutes, and finally observed under a microscope.

#### Animal studies

Female 4- to 6-week-old BALB/c nude mice were used to conduct all the *in vivo* studies. The mice were obtained from Shanghai Model Organisms and housed in a temperature-controlled room with free access to food and water under a natural day/night cycle.

For the xenograft model, the mice were subcutaneously inoculated in the right flank with HL7721 cells (5 × 10^5^), which had been knocked out for *NANOG*, *DDX4*, *PRDM1*, *SOX17*, *ACVR1 or SMAD5* using CRISPR-Cas9 technology. At the same time, HL7721-cKO-guide cells were inoculated as controls. Tumor dimensions were monitored using digital calipers and the time of tumor initiation was recorded. Upon completion of the experiments, the mice were humanely euthanized with 60% carbon dioxide for 5 minutes. Subsequently, the tumors were excised and collected for subsequent analysis.

#### Histology and HE staining

After obtaining tumors from BALB/c nude mice, the tissues were fixed overnight in 10% Neutral-Buffered Formalin (NBF) to preserve tissue integrity. Following fixation, the tissues were processed using standard paraffin embedding and sectioning protocols. Formalin-fixed, paraffin-embedded sections were used for subsequent hematoxylin-eosin (HE) staining. For HE staining, sections were deparaffinized, rehydrated, stained with hematoxylin-eosin using standard protocols.

#### Immunohistochemistry

For immunohistochemistry, 4 μm sections were deparaffinized, rehydrated, and subjected to antigen retrieval with sodium citrate buffer (Biotend, RE202004) via microwave heating. After blocking endogenous peroxidase with 3% H_2_O_2_ and non-specific binding with 5% BSA, sections were incubated overnight at 4°C with primary antibodies against OCT4 (Abcam, aab19857) and SOX2 (Abcam, ab171380). Immunodetection was performed using the Immunohistochemistry (IHC) Detect Kit for Rabbit/Mouse Primary Antibody (Proteintech, PK10006) according to the manufacturer’s instructions. Signals were visualized with DAB, counterstained with hematoxylin (Solarbio, G1004), and mounted with neutral balsam (Beyotime, C0173). Negative controls used PBS instead of primary antibodies. Images were captured via light microscopy.

### Quantification and statistical analysis

Data are presented as mean ± standard deviation (SD) and derived from at least three independent experiments. The unpaired t-test was used to compare differences between two groups, and one-way analysis of variance (ANOVA) was used to compare differences among multiple groups. Tumor-free survival curves were plotted using the Kaplan-Meier method, and differences were analyzed by the log-rank test. A *p* value <0.05 was considered statistically significant. ∗*p* < 0.05, ∗∗*p* < 0.01, ∗∗∗*p* < 0.001, ∗∗∗∗*p* < 0.0001; n. s., no significant statistical difference.

## Supplemental information

Document S1. Figures S1–S7 and Tables S1–S14

Table S15. Source data for PCA coordinates

Table S16. Source data for Euclidean distance

Table S17. Source data for the quadrant correlation analysis
