# Supplementary material for: Lineage tracing of soma-to-primordial germ cell-like conversion in human tumor cell line
Source: iScience. 2026 Jun 4;29(6):116214. doi: 10.1016/j.isci.2026.116214 (PMC13254981; doi:10.1016/j.isci.2026.116214)
Supplement: Document S1. Figures S1–S7 and Tables S1–S14 [file mmc1.pdf]

iScience, Volume *29*

Supplemental information

Lineage tracing of soma-to-primordial germ cell-like conversion in human tumor cell line

Jing Li, Fengyu Zhang, Ji Xiong, Aiping Liu, Peipei Liu, Zhan Ma, Hui-Kuan Lin, and Chunfang Liu

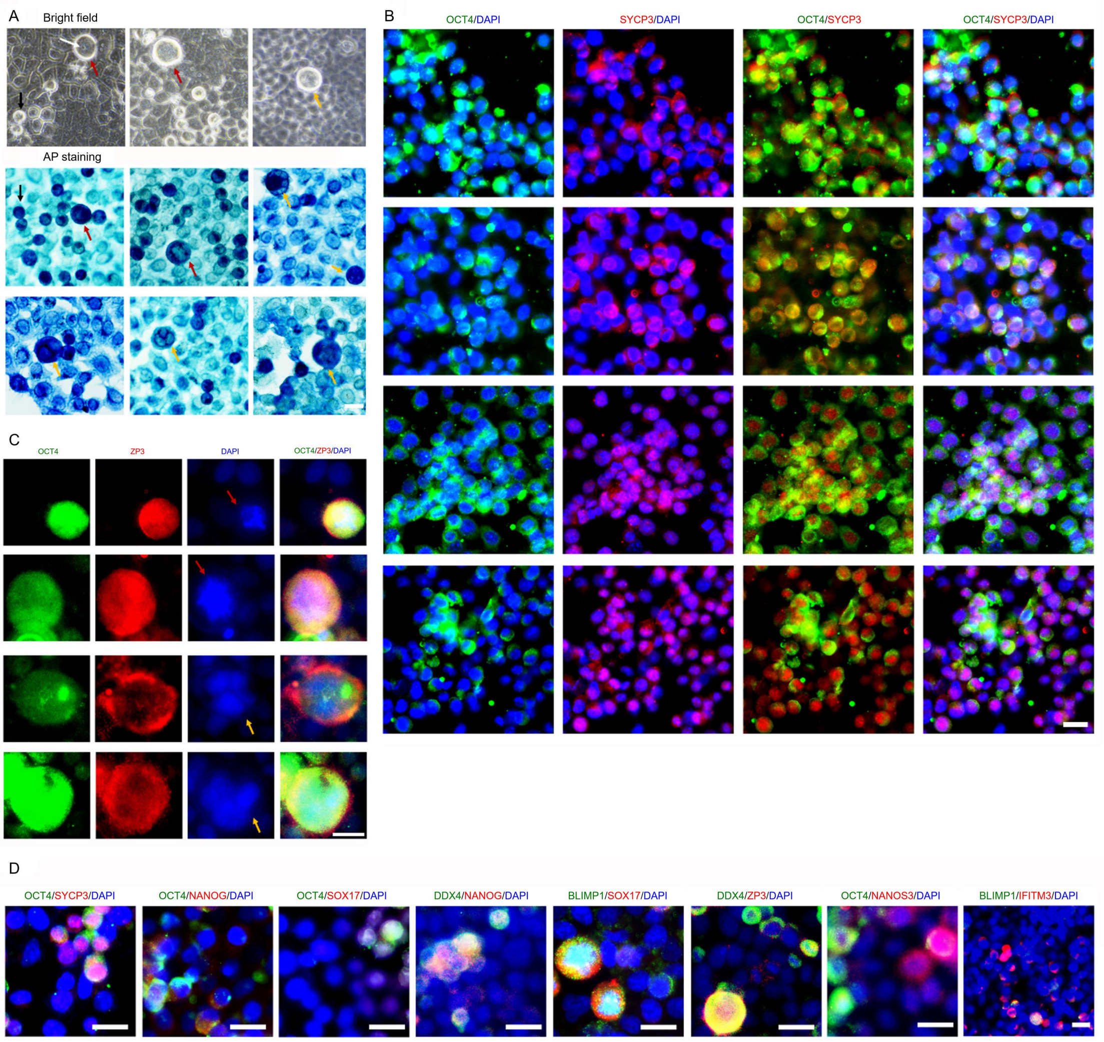

Figure S1. Generation of PGC-like cells in HL7721 cells, related to Figure 1. (A) Representative bright-field and AP staining images of germ cell-like cells at different developmental stages in HL7721 cells (PGC-like cells, black arrow; oocyte-like cells, red arrow; GV-like structure, white arrow; blastomere-like cells, yellow arrow). Scale bars, 25 µm. (B) Immunofluorescence showed that the germ cell-like cells were at the developmental stages based on the expression of OCT4 and SYCP3 in the HL7721 cultures. Scale bars, 25 µm. (C) Immunofluorescence showed the expression of OCT4 and ZP3 in the oocyte-like cells (red arrow) and blastomere-like cells (yellow arrow) in HL7721 cultures. Scale bars, 25 µm. (D) Immunofluorescence showed the expression of indicated proteins in weakly positive regions of HL7721 cultures. Scale bars, 25 µm.

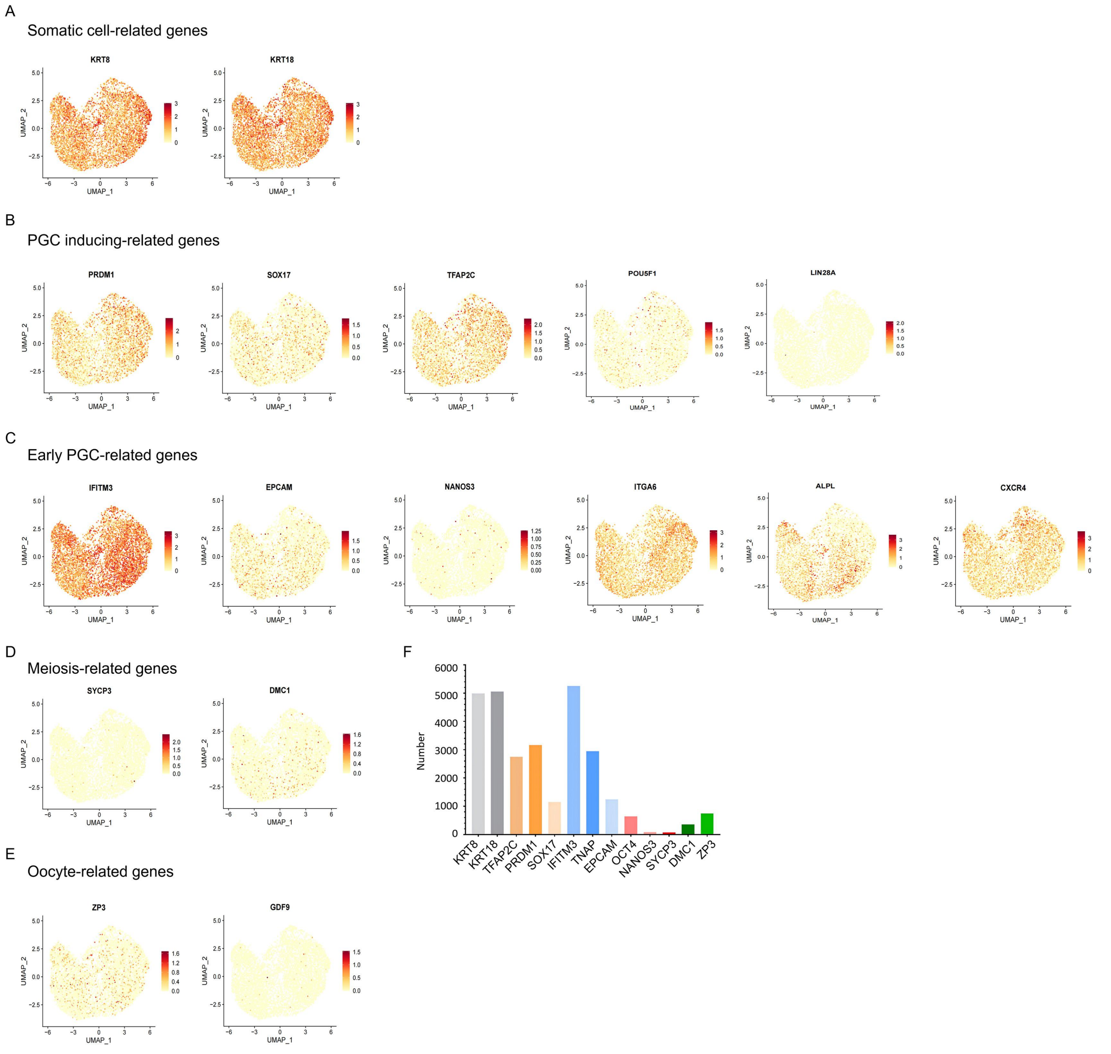

Figure S2. UMAP visualization of expression profiles, related to Figure 3. (A-E) UMAP visualization of the expression profiles of the indicated genes. (F) Quantification of the number of cells with high expression of the indicated genes.

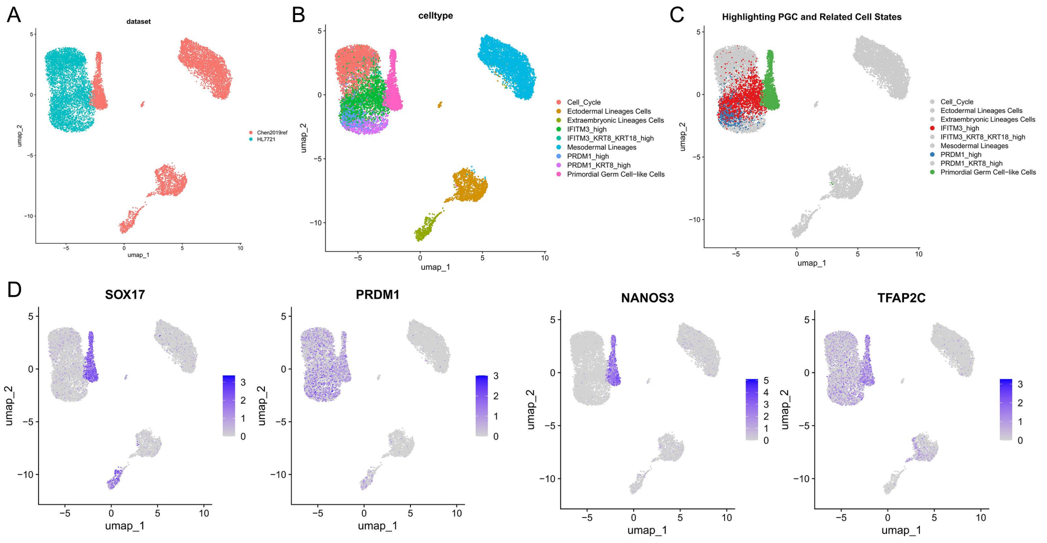

Figure S3. Integrated scRNA-seq analysis and marker gene expression of HL7721 and hPGCLC datasets, related to Figure 3. (A-C) UMAP visualization of integrated scRNA-seq datasets (including HL7721 and hPGCLC datasets), with cells colored by cell type annotation. PGC-like and related cell states are highlighted in the integrated UMAP plot. (D) Expression of PGC marker genes (*NANOS3*, *TFAP2C*, *SOX17*, *PRDM1*) in the integrated UMAP space.

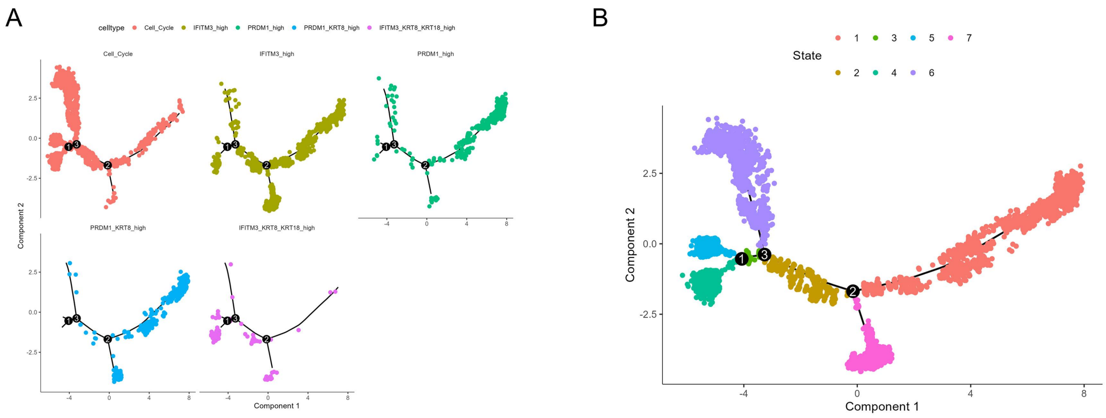

Figure S4. Pseudotime trajectory analysis of HL7721 cells, related to Figure 3D. (A) Pseudotime trajectories of individual cell subpopulations. (B) Cell state distribution along the pseudotime trajectory.

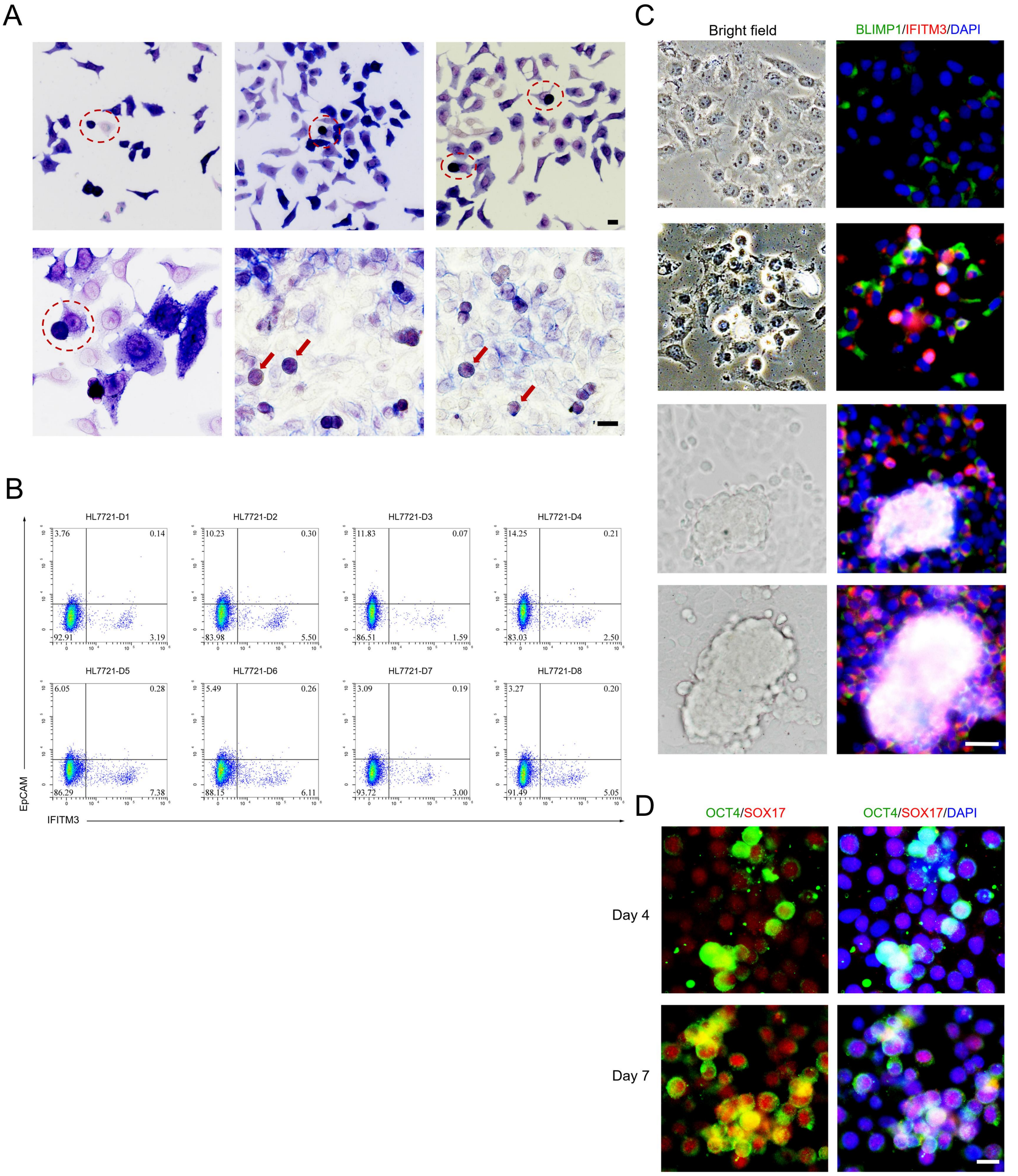

Figure S5. Derivation of PGC-like cells from somatic cells, related to Figure 5. (A) Bright field images showed AP staining for the possible nascent PGC-like cells in HL7721 cultures. Scale bars, 25 µm. Red circles indicate mutually attached somatic-like cells and PGC-like cells, while red arrows mark nascent PGC-like cells. (B) FCM results showed the ratio changes of IFITM3^{high} cells and EPCAM^{high} cells with the culture time extension. (C) Bright field and immunofluorescence images showed the morphology and the expression of indicated protein in HL7721 cultures. Scale bars, 100 µm. (D) Immunofluorescence showed the expression of OCT4 and SOX17 in HL7721 cultures at the different time points.

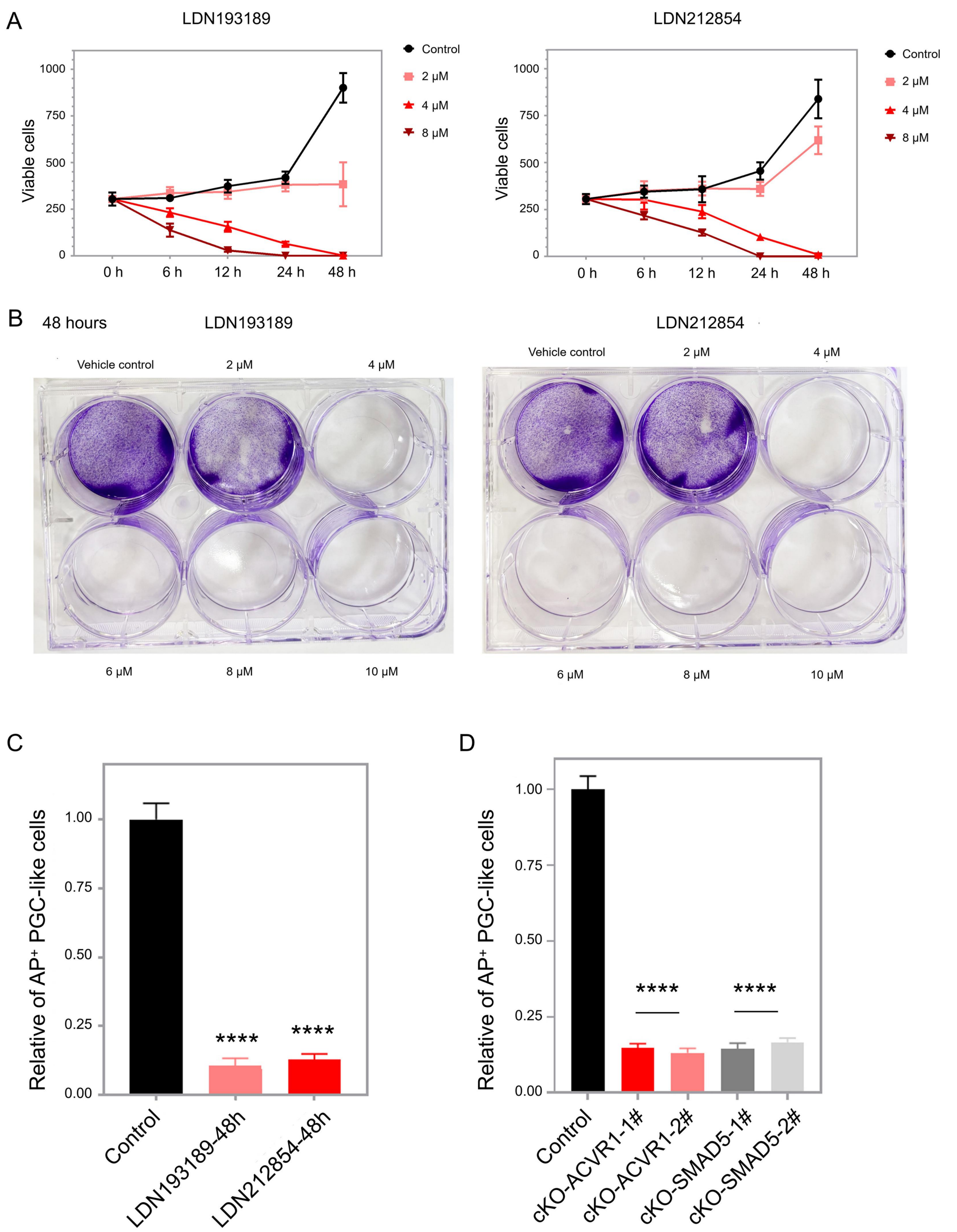

Figure S6. Roles of BMP pathway in SPLC, related to Figure 6 & 7. (A) Quantification of viable cells after treatment with DMSO (control), LDN193189 and LDN212854 respectively at different time points in HL7721 cultures (n = 10). (B) Viable cells stained with crystal violet of HL7721 cultures after treatment with DMSO (control), LDN193189 or LDN212854 for 48 h. (C) The plot showed the difference in AP-positive cell formation ability between the control group and the treatment group with LDN193189 or LDN212854 for 48 hours (n = 6). (D) The plot showed the difference of AP-positive cell formation ability in control group versus the indicated gene knockout group (n = 6). Data are presented as mean ± SD and analyzed by unpaired t test. *$p < 0.05$, **$p < 0.01$, ***$p < 0.001$, ****$p < 0.0001$; n. s., no significant statistical difference.

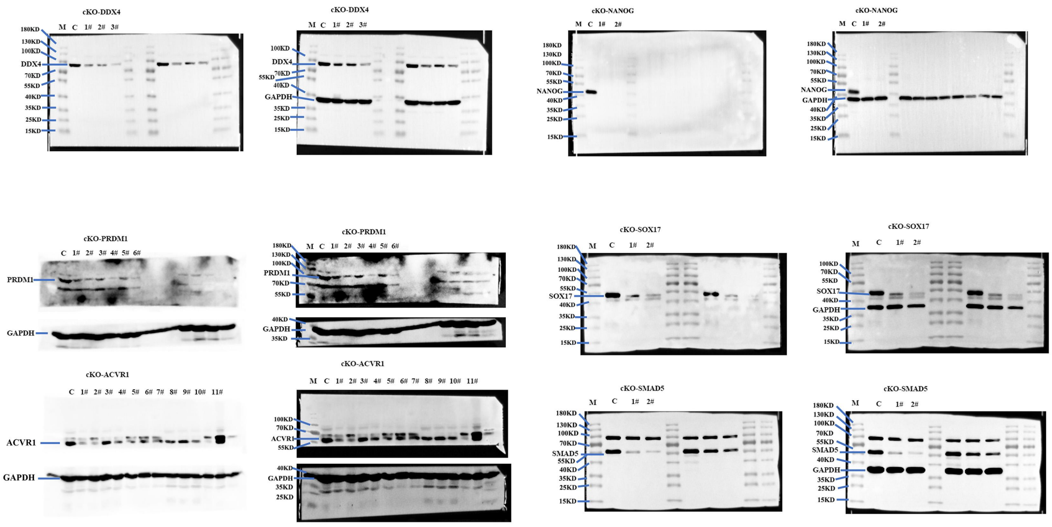

Figure S7. Images of the original western blot, related to Figure 2B, 6B and 7E.

Table S1. FACS results of HL7721, related to Figure 1C.

	HL7721(%)	
	Mean	SD
**IFITM3^{-} EpCAM^{+}**	28.94	0.39
**IFITM3^{+} EpCAM^{-}**	10.35	0.33
**IFITM3^{+} EpCAM^{+}**	2.71	0.13
**IFITM3^{+}**	13.05	0.45
**EpCAM^{+}**	31.64	0.38

Table S2. FACS results showed the difference of PGC-like cells in HL7721-Upper & Bottom, related to Figure 1G.

	HL7721-Upper (%)		HL7721-Bottom (%)		Upper & Bottom	
	**Mean**	**SD**	**Mean**	**SD**	**P value**	**P summary**
**IFITM3^{-} EpCAM^{+}**	45.84	0.21	12.28	0.21	<0.0001	****
**IFITM3^{+} EpCAM^{-}**	7.10	0.28	2.03	0.14	<0.0001	****
**IFITM3^{+} EpCAM^{+}**	8.07	0.09	0.13	0.04	<0.0001	****
**IFITM3^{+}**	15.17	0.27	2.16	0.17	<0.0001	****
**EpCAM^{+}**	53.91	0.14	12.41	0.21	<0.0001	****

Table S3. Relative RNA expression of indicated genes in HL7721-Upper & Bottom, related to Figure 1H.

	OCT4	***SOX2***	***NANOG***	***PRDM1***	***SOX17***	***TFAP2C***	***IFITM3***	***STELLAR***	***NANOS3***	***SYCP3***
**Mean**	1374.3	56.9	6290.6	705.1	614.7	45.3	156.0	1221.7	157.9	214.0
**SD**	66.4	12.6	160.0	156.0	287.4	9.7	34.2	394.4	9.8	26.0
**P value**	<0.0001	<0.0001	<0.0001	<0.0001	<0.0001	<0.0001	<0.0001	<0.0001	<0.0001	<0.0001
**P value summary**	****	****	****	****	****	****	****	****	****	****

Table S4. Relative number of AP^{+} PGC-like cells in HL7721 cKO group & control, related to Figure 1D, 6D and S6D.

	**Control**	**KO-*ACVR1*-1#**	**KO-*ACVR1*-2#**	**KO-*SMAD5*-1#**	**KO-*SMAD5*-2#**	**KO-*DDX4*-1#**	**KO-*DDX4*-2#**	**KO-*NANOG*-1#**	**KO-*NANOG*-2#**	**KO-*SOX17*-1#**	**KO-*SOX17*-2#**	**KO-*PRDM1*-1#**	**KO-*PRDM1*-1#**
**Mean**	1	0.143	0.131	0.145	0.152	0.05	0.037	0.042	0.038	0.12	0.105	0.082	0.103
**SD**	0.043	0.023	0.015	0.019	0.026	0.021	0.012	0.012	0.012	0.028	0.024	0.075	0.038
**P value**		<0.0001	<0.0001	<0.0001	<0.0001	<0.0001	<0.0001	<0.0001	<0.0001	<0.0001	<0.0001	<0.0001	<0.0001
**P value summary**		****	****	****	****	****	****	****	****	****	****	****	****

Table S5. Relative expression of RNA in cKO group & Control, related to Figure 2E, 6E and 7G.

		OCT4	*SOX2*	*NANOG*	*PRDM1*	*SOX17*	*TFAP2C*	*IFITM3*	*STELLAR*	*NANOS3*	*SYCP3*	*ZP3*
**cKO-*ACVR1*-1#**	**Mean**	0.0232	0.0610	0.0632	0.3269	0.0228	0.0706	0.0560	0.0701	0.2096	0.0016	0.0724
	**SD**	0.0052	0.0189	0.0628	0.0218	0.0006	0.0058	0.0051	0.0016	0.0735	0.0000	0.0047
	**Pvalue**	0.0070	<0.0001	0.0070	0.0256	<0.0001	<0.0001	<0.0001	<0.0001	0.0137	0.0040	<0.0001
	**P value summary**	**	****	**	*	****	****	****	****	*	**	****
**cKO-*ACVR1*-2#**	**Mean**	0.0086	0.0204	0.0079	0.7310	0.1109	0.1756	0.0531	0.0194	0.0951	0.0191	0.1204
	**SD**	0.0017	0.0056	0.0046	0.0864	0.0163	0.0165	0.0050	0.0052	0.0902	0.0298	0.0320
	**P value**	0.0067	<0.0001	0.0054	0.1022	<0.0001	<0.0001	<0.0001	<0.0001	0.0092	0.0044	<0.0001
	**P value summary**	**	****	**	ns	****	****	****	****	**	**	****
**cKO-*SMAD5*-1#**	**Mean**	0.0108	0.0115	0.0146	0.3219	0.1362	0.1454	0.0385	0.0189	0.0863	0.0012	0.0495
	**SD**	0.0013	0.0057	0.0176	0.0112	0.0100	0.0313	0.0023	0.0058	0.0573	0.0000	0.0017
	**Pvalue**	0.0067	<0.0001	0.0056	0.0232	<0.0001	<0.0001	<0.0001	<0.0001	0.0082	0.0040	<0.0001
	**P value summary**	**	****	**	*	****	****	****	****	**	**	****
**cKO-*SMAD5*-2#**	**Mean**	0.0137	0.0261	0.0442	0.3523	0.2461	0.1996	0.0698	0.0487	0.0523	0.0975	0.2168
	**SD**	0.0022	0.0161	0.0515	0.0471	0.0507	0.0199	0.0166	0.0087	0.0182	0.0983	0.0038
	**Pvalue**	0.0068	<0.0001	0.0064	0.0572	0.0001	<0.0001	<0.0001	<0.0001	0.0070	0.0069	<0.0001
	**P value summary**	**	****	**	ns	***	****	****	****	**	**	****
**cKO-*SOX17*-1#**	**Mean**	0.1131	0.0974	0.1162	0.2391	0.1397	0.2149	0.0534	0.1943	0.0456	0.0009	0.1007
	**SD**	0.0255	0.0275	0.0675	0.0181	0.0267	0.0144	0.0026	0.0151	0.0079	0.0000	0.0012
	**Pvalue**	0.0098	<0.0001	<0.0001	<0.0001	<0.0001	<0.0001	<0.0001	<0.0001	0.0068	0.0040	<0.0001
	**P value summary**	**	****	****	****	****	****	****	****	**	**	***
**cKO-*SOX17*-2#**	**Mean**	0.0969	0.0755	0.0907	1.4380	0.7077	0.2348	0.1546	0.1205	0.4316	0.0312	0.3001
	**SD**	0.0173	0.0341	0.0798	0.0375	0.1398	0.0550	0.0016	0.0203	0.0143	0.0307	0.0322
	**Pvalue**	0.0092	<0.0001	<0.0001	<0.0001	<0.0001	<0.0001	<0.0001	<0.0001	0.0108	0.0046	0.0002
	**P value summary**	**	****	****	****	****	****	****	****	*	**	***
**cKO-*NANOG*-1#**	**Mean**	0.0048	0.0176	0.0216	1.3020	0.9313	1.4563	0.1693	0.0058	0.1407	0.0112	0.0714
	**SD**	0.0006	0.0082	0.0257	0.1387	0.0585	0.2047	0.0495	0.0012	0.0152	0.0086	0.0084
	**Pvalue**	0.0066	<0.0001	0.0057	0.0655	0.1077	<0.0001	<0.0001	<0.0001	0.0097	0.0042	<0.0001
	**P value summary**	**	****	**	ns	ns	****	****	****	**	**	****

**cKO-*NANOG*-2#**	**Mean**	0.0180	0.1628	0.0859	1.1013	1.1534	0.9150	0.0144	0.0186	0.1774	0.0558	0.1893
	**SD**	0.0044	0.0329	0.0184	0.2185	0.1858	0.0683	0.0008	0.0033	0.0414	0.0270	0.0197
	**Pvalue**	0.0069	<0.0001	0.0072	0.0018	0.0113	<0.0001	<0.0001	<0.0001	0.0114	0.0050	<0.0001
	**P value summary**	**	****	**	ns	ns	****	****	****	*	**	****
**cKO-*DDX4*-1#**	**Mean**	0.0095	0.0042	0.0187	0.8563	1.1417	0.7054	0.1773	0.0030	0.3294	0.0878	0.1095
	**SD**	0.0065	0.0017	0.0140	0.0722	0.0862	0.1077	0.0068	0.0013	0.0979	0.0659	0.0047
	**Pvalue**	0.0067	<0.0001	0.0056	0.0175	0.0021	<0.0001	<0.0001	<0.0001	0.0242	0.0060	<0.0001
	**P value summary**	**	****	**	ns	ns	****	****	****	*	**	****
**cKO-*DDX4*-2#**	**Mean**	0.0047	0.0202	0.0236	0.8417	0.5631	0.4533	0.1243	0.0025	0.1048	0.0140	0.1015
	**SD**	0.0056	0.0111	0.0327	0.0338	0.4360	0.0933	0.0089	0.0003	0.0498	0.0202	0.0023
	**Pvalue**	0.0066	<0.0001	0.0058	0.0127	0.8219	<0.0001	<0.0001	<0.0001	0.0087	0.0042	<0.0001
	**P value summary**	**	****	**	ns	**	****	****	****	**	**	****
**cKO-*PRDM1*-1#**	**Mean**	0.0022	0.0004	0.0062	0.0607	0.0433	0.0236	0.0286	0.0024	0.0231	0.0025	0.0190
	**SD**	0.0010	0.0000	0.0055	0.0174	0.0069	0.0014	0.0066	0.0019	0.0283	0.0000	0.0038
	**Pvalue**	0.0065	<0.0001	0.0054	<0.0001	<0.0001	<0.0001	<0.0001	<0.0001	0.0063	0.0040	<0.0001
	**P value summary**	**	****	**	****	****	****	****	****	**	**	****
**cKO-*PRDM1*-2#**	**Mean**	0.0020	0.0025	0.0079	0.1458	0.0351	0.0058	0.0407	0.0039	0.1246	0.0351	0.0205
	**SD**	0.0010	0.0028	0.0073	0.0174	0.0148	0.0003	0.0014	0.0024	0.1546	0.0554	0.0013
	**Pvalue**	0.0065	<0.0001	0.0054	<0.0001	<0.0001	<0.0001	<0.0001	<0.0001	0.0126	0.0048	<0.0001
	**P value summary**	**	****	**	****	****	****	****	****	*	**	****
		OCT4	***SOX2***	***NANOG***	***PRDM1***	***SOX17***	***TFAP2C***	***IFITM3***	***STELLAR***	***NANOS3***	***SYCP3***	***ZP3***

Table S6. The difference of AP+ PGC-like cells at different time points of HL7721 culture, related to Figure 5B.

	**Day1**		**Day3**		**Day5**		**Day7**	
	Mean	SD	Mean	SD	Mean	SD	Mean	SD
	1	0.07	1.505	0.14	24.65	2.21	51.23	5.01
	**D1&D3**	**D1&D5**	**D1&D7**	**D3&D5**	**D3&D7**	**D5&D7**		
**P value**	<0.0001	<0.0001	<0.0001	<0.0001	<0.0001	<0.0001		
**P value summary**	****	****	****	****	****	****		

Table S7. FACS results showed the difference of PGC-like cells at different time points of HL7721 culture, related to Figure 5C & S5B.

	Day1		Day2		Day3		Day4		Day5		Day6		Day7		Day8	
	Mean	SD	Mean	SD	Mean	SD	Mean	SD	Mean	SD	Mean	SD	Mean	SD	Mean	SD
IFITM3⁻ EpCAM⁺	3.95	0.23	10.60	0.45	11.76	0.19	14.27	0.16	6.07	0.16	5.53	0.15	3.28	0.34	3.26	0.08
IFITM3⁺ EpCAM⁻	3.37	0.16	5.43	0.15	1.54	0.04	2.28	0.21	6.92	0.43	6.42	0.32	2.70	0.27	4.99	0.16
IFITM3⁺ EpCAM⁺	0.12	0.02	0.21	0.09	0.07	0.01	0.21	0.03	0.24	0.05	0.29	0.12	0.15	0.04	0.29	0.08
IFITM3⁺	3.49	0.14	5.64	0.22	1.61	0.05	2.49	0.20	7.16	0.48	6.71	0.41	2.85	0.31	5.28	0.16
EpCAM⁺	4.07	0.22	10.81	0.42	11.83	0.19	14.48	0.19	6.31	0.13	5.82	0.07	3.44	0.31	3.55	0.11

		P value	Sum		P value	Sum		P value	Sum	
IFITM3+	D1 vs. D2	<0.0001	****	D2 vs. D5	0.0001	***	D4 vs. D5	<0.0001	****	
	D1 vs. D3	<0.0001	****	D2 vs. D6	0.0046	**	D4 vs. D6	<0.0001	****	
	D1 vs. D4	0.0084	**	D2 vs. D7	<0.0001	****	D4 vs. D7	0.7549	ns	
	D1 vs. D5	<0.0001	****	D2 vs. D8	0.7549	ns	D4 vs. D8	<0.0001	****	
	D1 vs. D6	<0.0001	****	D3 vs. D4	0.0233	*	D5 vs. D6	0.508	ns	
	D1 vs. D7	0.1603	ns	D3 vs. D5	<0.0001	****	D5 vs. D7	<0.0001	****	
	D1 vs. D8	<0.0001	****	D3 vs. D6	<0.0001	****	D5 vs. D8	<0.0001	****	
	D2 vs. D3	<0.0001	****	D3 vs. D7	0.0011	**	D6 vs. D7	<0.0001	****	
	D2 vs. D4	<0.0001	****	D3 vs. D8	<0.0001	****	D6 vs. D8	0.0002	***	

Table S8. Efficiency of generation of PGC-like cells from HL7721 cells at the single cell level.

Total single cells	Clones (>64cells)	Clones with PGC-like cells	Efficiency of generation of PGC-like cells
66	51	51	75%

Table S9. Relative RNA expression of indicated genes in different time points of HL7721 culture, related to Figure 5E.

	6h		24h		48h		Day3		Day7		Day10				
	Mean	SD	Mean	SD	Mean	SD	Mean	SD	Mean	SD	Mean	SD		P Value	Sum
SOX2	1.043	0.354	3.651	0.372	0.480	0.138	0.836	0.103	7.269	0.750	41.768	5.743		0.0005	***
IFITM3	1.001	0.058	0.947	0.036	1.452	0.035	0.735	0.439	1.222	0.086	0.437	0.041		0.0001	***
NANOG	1.051	0.377	1.929	0.294	0.213	0.116	0.361	0.147	8.071	1.767	23.445	3.745		0.0030	**
PRDM1	1.005	0.115	0.535	0.082	0.640	0.083	0.563	0.114	0.805	0.121	2.518	0.125		<0.0001	****

STELLAR	1.003	0.099	1.912	0.609	0.124	0.018	0.378	0.047	3.820	0.610	3.392	0.437		0.3797	ns
SOX17	1.003	0.096	0.548	0.026	0.002	0.000	0.526	0.072	2.333	0.244	0.200	0.036		0.0001	***
POU5F1	1.009	0.163	2.261	0.069	0.004	0.004	0.703	0.355	9.036	0.560	18.765	0.327		<0.0001	****
NANOS3	1.001	0.041	0.911	0.409	1.189	0.355	0.346	0.142	1.845	0.652	1.727	1.181		0.8875	ns
TFAP2C	1.004	0.103	2.498	0.117	3.826	0.421	1.4776	0.321	2.885	0.269	0.805	0.081		0.0001	***
		**6h&24h**	**6h&48h**	**6h&D3**	**6h&D7**	**6h&D10**	**24h&48h**	**24h&D3**	**24h&D7**	**24h&D10**	**48h&D3**	**48h&D7**	**48h&D10**	**D3&D7**	**D3&D10**
SOX2	**P value**	0.0009	0.0620	0.348	0.0002	0.0003	0.0002	0.0002	0.0017	0.0003	0.0232	0.0001	0.0002	0.0001	0.0002
	**Sum**	***	ns	ns	***	***	***	***	**	***	*	***	***	***	***
IFITM3	**P value**	0.2436	<0.0001	0.3559	0.0207	0.0002	<0.0001	0.4498	0.0070	<0.0001	0.1774	<0.0001	0.0078	0.1319	0.3072
	**Sum**	ns	****	ns	*	***	****	ns	**	****	ns	****	**	ns	ns
NANOG	**P value**	0.0335	0.0210	0.0415	0.0025	0.0005	0.0007	0.0012	0.0040	0.0006	0.2422	0.0015	0.0004	0.0017	0.0004
	**Sum**	*	*	*	**	***	***	***	**	***	ns	**	***	**	***
PRDM1	**P value**	0.0045	0.0112	0.0091	0.1072	0.0001	0.1937	0.7477	0.0329	<0.0001	0.3979	0.1234	<0.0001	0.0654	<0.0001
	**Sum**	**	*	**	ns	***	ns	ns	*	****	ns	ns	****	ns	****
STELLAR	**P value**	0.0634	0.0001	0.0006	0.0014	0.0008	0.0071	0.0122	0.0186	0.0268	0.0010	0.0005	0.0002	0.0006	0.0003

	**Sum**	ns	***	***	**	***	**	*	*	*	***	***	***	***	***
SOX17	**P value**	0.0014	<0.0001	0.0023	0.0009	0.0002	<0.0001	0.6488	0.0002	0.0002	0.0002	<0.0001	0.0007	0.0002	0.0022
	**Sum**	**	****	**	***	***	****	ns	***	***	***	****	***	***	**
POU5F1	**P value**	0.0003	0.0004	0.2449	<0.0001	<0.0001	<0.0001	0.0017	<0.0001	<0.0001	0.0207	<0.0001	<0.0001	<0.0001	<0.0001
	**Sum**	***	***	ns	****	****	****	**	****	****	*	****	****	****	****
NANOS3	**P value**	0.7249	0.4134	0.0016	0.0888	0.3469	0.4244	0.0867	0.1035	0.3212	0.0189	0.2009	0.4917	0.0177	0.1146
	**Sum**	ns	ns	**	ns	ns	ns	ns	ns	ns	*	ns	ns	*	ns
TFAP2C	**P value**	0.2436	0.0003	0.0061	0.0207	0.0002	<0.0001	0.0080	0.0070	<0.0001	0.0004	0.0127	<0.0001	0.0019	0.2698
	**Sum**	ns	***	**	*	***	****	**	**	****	**	*	****	**	ns
		**6h&2h**	**6h&48h**	**6h&D3**	**6h&D7**	**6h&D10**	**24h&48h**	**24h&D3**	**24h&D7**	**24h&D10**	**48h&D3**	**48h&D7**	**48h&D10**	**D3&D7**	**D3&D10**

Table S10. Relative number of AP^+ PGC-like cells in 7721 cultures after treated with DMSO (control) or LDN193189 or LDN212854 for 48 hours, related to Figure S6C.

	**DMSO**	**LDN193189**	**LDN212854**
**Mean**	1	0.106	0.129
**SD**	0.059	0.027	0.021
**P value**	<0.0001	<0.0001	<0.0001
**P value summary**	****	****	****

Table S11. Primers for qRT-PCR and nested PCR.

**Gene**	**Forward/reverse**	**Sequence 5'-3'**
hqOCT4	forward	GCTGGAGCAAAACCCGGAGG
	reverse	TCGGCCTGTGTATATCCCAGGGTG
hqSOX2	forward	GGGAAATGGGAGGGGTGCAAAAGAGG
	reverse	TTGCGTGAGTGTGGATGGGATTGGTG
hqNANOG	forward	TGCTGAGATGCCTCACACGGA

	reverse	TGACCGGGACCTTGTCTTCCTT
hqIFITM3	forward	TGTCCAAACCTTCTTCTCTCC
	reverse	CGTCGCCAACCATCTTCC
hqSTELLAR	forward	ACGCCGATGGACCCATCACAGTTT
	reverse	TCTCGGAGGAGATTTGAGAGGCCC
hqNANOS3	forward	ACAGCCACACCACCCGAAACT
	reverse	GGCTCAGACTTCCCGGCACCT
hqSCP3	forward	CTAGAATTGTTCAGAGCCAGAGA
	reverse	GTTCAAGTTCTTTCTTCAAAG
hqBLIMP1	forward	CGGGGAGAATGTGGACTGGGTAGAG
	reverse	CTGGAGTTACACTTGGGGGCAGC
hqSOX17	forward	CGGGGAGAATGTGGACTGGGTA
	reverse	CTGGAGTTACACTTGGGGGCAGC
hqTFAP2C	forward	GTTCTCAGAAGAGCCAAATCGAA
	reverse	CGGCTTCACAGACATAGGCAA
hqZP3	forward	GAGGCAGCCTCATGTCATG
	reverse	AGGCAAAGCCCACTGCTC
hqGADPH	forward	CAAAGTTGTCATGGATGACC
	reverse	CCATGGAGAAGGCTGGGG
hqTNAP	forward	AAGCAGGTCTTGGGGTGCACCA
	reverse	TTGGTCTCGCCAGTACTTGGGGT
hH19DMR	outside forward	TTTTTGGTAGGTATAGAGTT
	outside reverse	AAACCATAACACTAAAACCC
	inside forward	TGTATAGTATATGGGTATTTTTGGAGGTTT
	inside reverse	TCCCATAAATATCCTATTCCCAAATAACC

Table S12. sgRNAs sequences (Sigma).

**Ensemble Symbol**	**gene_id**	**Clone ID**	**seq target with PAM**
DDX4	ENSG00000152670	HS5000003233	CTTGGAAGTCAGAAGCAGAAGG
DDX4	ENSG00000152670	HS5000003234	TGAAATGATCTCTTCGAGAAGG
NANOG	ENSG00000111704	HS5000008214	GAGAAGAGTGTCGCAAAAAAGG
PRDM1	ENSG00000057657	HS5000027303	AAGTGGTGAAGCTCCCCTCTGG
PRDM1	ENSG00000057657	HS5000027304	TAATGAAGAGAAAAGCAACTGG
SOX17	ENSG00000164736	HS5000023939	AGCCCCATCGGGGACATGAAGG
ACVR1	ENSG00000115170	HS5000009497	CTGGGAATGCATGACTGCCAGG
ACVR1	ENSG00000115170	HS5000009498	GTGGGGAACAATCCCCGTGTGG
SMAD5	ENSG00000113658	HS5000009752	GAAGATATGGGGTTCAGAGGGG

Table S13. Sequencing data of knockout cells generated by CRISPR-Cas9 technology, related to Figure 2B, 6B and 7E.

Genes	Sequencing
**HL7721-cKO-*DDX4*-1#**	Query 483 AATGGAGACACTTTTCA-A-GACATCTCCTCCTTCAGGGATGTGTGTAAAGCGGCATGGC 540<br>Sbjct 112644545 AATGGAGACACTTTTAACAGGACTTCAGCTTCATCAGGTATGTGTTTAAGGCAAGATGGC 112644486<br>sgRNA: AATGGAGACACTTTTAACAGG **Del: 2bp**
**HL7721-cKO-*DDX4*-2#**	Query 481 AATGGAGACACTTT-AACACGACTTCTTCTTCATCAAGGATGTGTTTAAAGGAAGATGGT 539<br>Sbjct 112644545 AATGGAGACACTTTTAACAGGACTTCAGCTTCATCAGGTATGTGTTTAAGGCAAGATGGC 112644486<br>sgRNA: AATGGAGACACTTTTAACAGG Del:1bp
**HL7721-cKO-*NANOG*-1#**	Query 108 CCTGATTCTTCTACCAGTCCCAAA--CAAA-AGCTCTCAAGTCCTGAGGCTGACAAG-G- 162<br>Sbjct 7792994 CCTGATTCTTCCACCAGTCCCAAAGGCAAACAAC-C-C-ACTTCTGCAG-AGAAGAGTGT 7793049<br>Query 163 CCCTGAGGAGGAGGAGAACAAGGTCCTTGCCAGGAAGCAGAAGATGCGGACTGTGTTCTC 222<br>Sbjct 7793050 CGCAAAAAAGGA--AG-ACAAGGTCCCGGTCAAGAAACAGAAGACCAGAACTGTGTTCTC 7793106<br>sgRNA: GAGAAGAGTGTCGCAAAAAAGG **Int: 2bp**
**HL7721-cKO-*NANOG*-2#**	Query 182 ACAGCCCTGATTCTTCCACCAGTCCCAAAGGCAAACAACCCACTTCTGCAGAGAATAGTG 241<br>Sbjct 7792989 ACAGCCCTGATTCTTCCACCAGTCCCAAAGGCAAACAACCCACTTCTGCAGAGAAGAGTG 7793048<br>Query 242 --GCAAAAAAGGAAAACAAGGTCCCAGTCAAGAAACAGAAGACCAGAACTGTGTTCTCTT 299<br>Sbjct 7793049 TCGCAAAAAAGGAAGACAAGGTCCCGGTCAAGAAACAGAAGACCAGAACTGTGTTCTCTT 7793108<br>sgRNA: GAGAAGAGTGTCGCAAAAAAGG **Del: 2bp**
**HL7721-cKO-*PRDM1*-1#**	Query 121 CCAGAG-----CTTCACCACTTCATTGACGGCTTTAATGAAGAGAAAAGCAACTGGATGC 175<br>Sbjct 106099307 CCAGAGGGGAGCTTCACCACTTCATTGACGGCTTTAATGAAGAGAAAAGCAACTGGATGC 106099366<br>sgRNA: AAGTGGTGAAGCTCCCCTCTGG **Del: 5bp**
**HL7721-cKO-*PRDM1*-2#**	Query 122 GGGGAGCTTCACCACTTCATTGACGGCTTTAATGAAGAGAAAA----CTGGATGCGCTAT 177<br>Sbjct 106099312 GGGGAGCTTCACCACTTCATTGACGGCTTTAATGAAGAGAAAAGCAACTGGATGCGCTAT 106099371<br>sgRNA: TAATGAAGAGAAAAGCAACTGG **Del: 4bp**
**HL7721-cKO-*SOX17*-1#**	Query 122 GCCCGCGGTGATGGCCGGGCTGGGCCCCTGCCCCTGGGCCGAGTCGCTGAGCCCCATCGG 181<br>Sbjct 54458198 GCCCGCGGTGATGGCCGGGCTGGGCCCCTGCCCCTGGGCCGAGTCGCTGAGCCCCATCGG 54458257<br>Query 182 GGACAAGGAAAGGGAAAGGCGAGGCGCCAGCGAACAGCGCAGCACCGGGCGGGGCCGCGG 241<br>Sbjct 54458258 GGACATG-AAGGTGAAGGGCGAGGCGCCGGCGAACAGCGGAGCACCGGCCGGGGCCGCGG 54458316<br>sgRNA:AGCCCCATCGGGGACATGAAGG **Int: 1bp**

<table>
<tr><td>HL7721-cKO-SOX17-2#</td><td>Query 122 CTGCCCGCGGTGATGGCCGGGCTGGGCCCCTGCCCCTGGGCCGAGTCGCTGAGCCCCATT 181
Sbjct 54458196 CTGCCCGCGGTGATGGCCGGGCTGGGCCCCTGCCCCTGGGCCGAGTCGCTGAGCCCCATC 54458255

Query 182 GGGG--------GAGAAGGGCGAGGCGCCGGCAAACAGCGGAGCACCGGCCGGGGCCGCG 233
Sbjct 54458256 GGGGACATGAAGGTGAAGGGCGAGGCGCCGGCGAACAGCGGAGCACCGGCCGGGGCCGCG 54458315
sgRNA: AGCCCCATCGGGGACATGAAGG Del: 8BP</td></tr>
<tr><td>HL7721-cKO-ACVR1-1#</td><td>Query 427 TTGATGTGGGG-----------------CACCAAGCGCTACATGGCCCCCGAAGTTCTAG 469
Sbjct 157761041 TTGATGTGGGGAACAATCCCCGTGTGGGCACCAAGCGCTACATGGCCCCCGAAGTTCTAG 157760982
sgRNA: TGGGGAACAATCCCCGTGTGG Del: 17BP</td></tr>
<tr><td>HL7721-cKO-ACVR1-2#</td><td>Query 363 GTGGGTTGATTACATTGTTATCTTCTTCCTTTCAGGCCTGGCAAGCCAGGCATTCCCAAA 422
Sbjct 157761112 GTGGGTTGATTACATTGTTATCTTCTTCCTTTCAGGCCTGGC-AGTCATGCATTCCCAGA 157761054
sgRNA: CTGGGAATGCATGACTGCCAGG Int: 1bp</td></tr>
<tr><td>HL7721-cKO-SMAD5-1#</td><td>Query 182 GGGCCTCTTCAGTGGCTGGATAAAGTCCTTAC-----------ACCTTCTG--------- 221
Sbjct 136177406 GGGCCTCTTCAGTGGCTGGATAAAGTCCTTACTCAGATGGGCTCCCCTCTGAACCCCATA 136177465
sgRNA GAAGATATGGGGTTCAGAGGGG Del: 22BP
Query 222 --TTTCGTTTCGCACAGCTAATCTTTTCTTTTCTATTGTTATGGTACTTGACTTGTTTTT 279
Sbjct 136177466 TCTTCTGTTTCATAATGCAGAAGTATTCTTTTCAATTATATTGTTAGTGGACTTGTTTTA 136177525</td></tr>
<tr><td>HL7721-cKO-SMAD5-2#</td><td>Query 179 ATTCATCTTCTTGGGCCTCTTCAGTGGCTGGATAAATTCCTTACTCAGATGGGCTCCCCT 238
Sbjct 136177394 ATTCATCTTCATGGGCCTCTTCAGTGGCTGGATAAAGTCCTTACTCAGATGGGCTCCCCT 136177453

Query 239 C-----------TCTTCGGTTTCGTGTCGCAGAACTAATCTTTTCTTTTATGTTGTAATG 287
Sbjct 136177454 CTGAACCCCATATCTTCTGTTTCATAATGCAGAAGTATTCTTTTCAATTATATTGTTAGT 136177513
sgRNA GAAGATATGGGGTTCAGAGGGG Del: 11bp</td></tr>
</table>

Table S14. Representative Sequencing Results of *H19*-DMR TA Cloning in HL7721, related to Figure 1F.

<table>
<tr><th>Clone</th><th>Sequencing</th></tr>
<tr><td>1#</td><td>Query 1 TCCCATAAATATCCTATTCCCAAATAACCCCCATAAACCCTACAACACATAACTTAAATA 60
Sbjct 192364 TCCCATGAGTGTCCTATTCCCAGATGACCCCCGTGAACCCTGCGACGCGTGGCTTGGGTG 192423

Query 61 ACCCAAAACATTTCCACGAACAAACCCCAATTAAAACAAACTCAAACTATAATATATAAA 120
Sbjct 192424 ACCCGGGACGTTTCCACGGGCGAACCCCAGTTGGGGCGGGCTCGGGCTGTGATGTGTGAG 192483

Query 121 CCTACACTACCACCACACAACCACTTCCAATTCCACAACTACAACCAATTCCATACCATC 180
Sbjct 192484 CCTGCACTGCCGCCGCGCGGCCACTTCCGATTCCACAACTACAACCAATTCCGTGCCATC 192543

Query 181 CAAACAATAAAACCGaaaaaaaaaCCTCCAAAAATACCCATATACTATACA 231
Sbjct 192544 CAGGCGGTGAGACCGAAGGAGAAGCCTCCAGAAATACCCATGTGCTATGCA 192594</td></tr>
</table>

2#

```
Query   1       TCCCATAAATATCCTATTCCCAAATAACCCCCATAAACCCTACAACACATAACTTAAATA  60
                |||||| | | ||||||||||| || |||||| | |||||| | || | |  |||   |
Sbjct   192364  TCCCATGAGTGTCCTATTCCCAGATGACCCCCGTGAACCCTGCGACGCGTGGCTTGGGTG  192423

Query   61      ACCCAAAACATTTCCACAAACAAACCCCAATTAAAACAAACTCAAACTATAATATATAAA  120
                ||||   || |||||||   | ||||||| ||    |   |||   || | || | | |
Sbjct   192424  ACCCGGGACGTTTCCACGGGCGAACCCCAGTTGGGGCGGGCTCGGGCTGTGATGTGTGAG  192483

Query   121     CCTACACTACCGCCACGCAACCACTTCCAATTCCACAACTACAACCAATTCCATACCATC  180
                ||| |||| ||||| |||  |||||||| ||||||||||||||||||||||| | |||||
Sbjct   192484  CCTGCACTGCCGCCGCGCGGCCACTTCCGATTCCACAACTACAACCAATTCCGTGCCATC  192543

Query   181     CAAACAATAAAACCGaaaaaaaaaCCTCCAAAAATACCCATATACTATACA  231
                ||  |  | | ||||||  | || |||||| |||||||||| | |||| ||
Sbjct   192544  CAGGCGGTGAGACCGAAGGAGAAGCCTCCAGAAATACCCATGTGCTATGCA  192594
```

3#

```
Query   1       TCCCATAAATATCCTATTCCCAAATAACCCCCATAAACCCTACGACGCATAACTTAAATA  60
                |||||| | | ||||||||||| || |||||| | |||||| |||||| |  |||   |
Sbjct   192364  TCCCATGAGTGTCCTATTCCCAGATGACCCCCGTGAACCCTGCGACGCGTGGCTTGGGTG  192423

Query   61      ACCCAAAACATTTCCACGAACGAACCCCAATTAAAACAAACTCGAACTATAATATATAAA  120
                ||||   || ||||||||  ||||||||| ||    |   ||||  || | || | | |
Sbjct   192424  ACCCGGGACGTTTCCACGGGCGAACCCCAGTTGGGGCGGGCTCGGGCTGTGATGTGTGAG  192483

Query   121     CCTACACTACCGCCACGCAACCACTTCCGATTCCACAACTACAACCAATTCCGTACCATC  180
                ||| |||| ||||| |||  |||||||||||||||||||||||||||||||||| |||||
Sbjct   192484  CCTGCACTGCCGCCGCGCGGCCACTTCCGATTCCACAACTACAACCAATTCCGTGCCATC  192543

Query   181     CAAACAATAAAACCGaaaaaaaaaCCTCCAAAAATACCCATATACTATACA  231
                ||  |  | | ||||||  | || |||||| |||||||||| | |||| ||
Sbjct   192544  CAGGCGGTGAGACCGAAGGAGAAGCCTCCAGAAATACCCATGTGCTATGCA  192594
```
